# Bayesian Meta-Analysis: Impacts of Eating Habits and Habitats on Omega-3 Long-Chain Polyunsaturated Fatty Acid Composition and Growth in Cultured Fish

**DOI:** 10.3390/ani14142118

**Published:** 2024-07-20

**Authors:** Yuanbing Wu, Ania Rashidpour, Isidoro Metón

**Affiliations:** Secció de Bioquímica i Biologia Molecular, Departament de Bioquímica i Fisiologia, Facultat de Farmàcia i Ciències de l’Alimentació, Universitat de Barcelona, Joan XXIII 27-31, 08028 Barcelona, Spain; wuuanbing@gmail.com (Y.W.); aniyarashidpoor2017@gmail.com (A.R.)

**Keywords:** vegetable oil, fatty acids, fish intermediary metabolism, meta-analysis, aquaculture

## Abstract

**Simple Summary:**

Simple Summary: Due to limited availability, fluctuating prices, and sustainability concerns, the aquaculture sector is increasingly replacing fish oil with vegetable oil in aquafeeds. To comprehensively explore the dependence of growth performance and omega-3 long-chain polyunsaturated fatty acid (*n*-3 LC-PUFA) composition on dietary *n*-3 LC-PUFA levels in cultured fish with varying feeding habits (herbivorous, omnivorous, and carnivorous) and habitats (marine and freshwater), we employed a Bayesian meta-analysis to quantitatively analyze data from 81 selected studies. This novel approach allowed us to show to what extent the growth performance and *n*-3 LC-PUFA tissue levels of freshwater and herbivorous fish exhibit higher tolerance than marine and carnivorous fish to reduced amounts of dietary *n*-3 LC-PUFA levels. The results of this study can help optimize the use of fish oil in aquafeeds and contribute to the development of more sustainable aquaculture practices.

**Abstract:**

Omega-3 long-chain polyunsaturated fatty acids (*n*-3 LC-PUFAs) such as eicosapentaenoic acid (20:5*n*-3, EPA) and docosahexaenoic acid (22:6*n*-3, DHA) offer protective benefits against various pathological conditions, including atherosclerosis, obesity, inflammation, and autoimmune diseases. Marine fish and seafood are the primary sources of *n*-3 LC-PUFAs in the human diet. However, the inclusion of fish oil in aquafeeds is declining due to limited availability, fluctuating prices, sustainability concerns, and replacement with vegetable oils. While comprehensive narrative reviews on the impact of substituting fish oil with vegetable oil in aquafeeds exist, quantitative studies are relatively scarce and mainly focused on comparing the source of vegetable oils. Herein, we employed, for the first time, a Bayesian meta-analysis approach, collecting research data from 81 articles to quantitatively analyze the effects of dietary *n*-3 LC-PUFA levels on the *n*-3 LC-PUFA composition and growth performance in cultured fish. Our findings indicate that with the exception of herbivorous fish, dietary *n*-3 LC-PUFA levels significantly affect the EPA and DHA levels in the livers and muscles of carnivorous, omnivorous, freshwater, and marine fish. Additionally, the growths of freshwater and herbivorous fish were less affected by changes in dietary *n*-3 LC-PUFA levels compared to that of carnivorous and marine fish.

## 1. Introduction

Evidence from clinical and laboratory research supports general beneficial effects of omega-3 long-chain polyunsaturated fatty acids (*n*-3 LC-PUFAs), especially eicosapentaenoic acid (EPA) and docosahexaenoic acid (DHA), on inflammation, cardiovascular diseases, and neural development, among others [1]. Linoleic acid and α-linolenic acid are precursors for the synthesis of *n*-6 and *n*-3 LC-PUFA series, respectively, and are essential dietary fatty acids for vertebrates, which lack the Δ12/*n*-6 and Δ15/*n*-3 desaturase activities required to synthesize linoleic acid from oleic acid and α-linolenic acid from linoleic acid. In addition, vertebrates, including bony fish, can only convert a small portion of linoleic acid and α-linolenic acid to LC-PUFAs and at insufficient rates to cover physiological demands. Therefore, LC-PUFAs such as EPA and DHA are essential components of a healthy diet. Fish is considered the main source of *n*-3 LC-PUFAs, such as EPA and DHA, in the human diet. Currently, some narrative reviews have well elucidated the importance of *n*-3 LC-PUFAs in fish and the related molecular biology reasons [2,3].

In marine fish aquaculture, fish oil produced from captures in fishing grounds is an ideal source of feed lipids. However, limited marine fishery resources make it challenging to meet the growing demand for fish oil in the aquaculture industry. Aquaculture production contributions to the total world fisheries and aquaculture increased from 32.3% in 2000 to 49.2% in 2020, among which the fish farmed in marine waters accounted for 37.8% of the total aquaculture production in 2020 [4]. The growing production of aquaculture is concomitant with the increasing manufacture of aquafeeds, which are mainly composed of 18–50% protein, 10–25% lipids, and 15–20% carbohydrates, depending on fish species [5]. However, the global capture fisheries production almost stopped growing since the mid-1980s due to sustainability limits. Hence, the growth of aquaculture has driven the aquafeed industry to substitute fish oil with more readily available and cost-effective sources, specifically vegetable oils and animal fats. These common lipids are abundant in linoleic acid but are devoid of *n*-3 LC-PUFAs such as EPA and DHA. Thus, this source transition may impact *n*-3 LC-PUFA composition and fish growth performance.

Based on the above-mentioned reasons, extensive efforts have been directed towards the substitution of fish oil with vegetable oil or terrestrial animal fat in aquafeeds. From the results of these studies, it is evident that both growth performance and *n*-3 LC-PUFA deposition in the liver and skeletal muscle are highly influenced by the source of dietary lipids. Differences in growth performances exhibited by fish species in a controlled artificial aquaculture environment primarily stem from inherent specific variations related to the synthesis capacity and physiological requirements of *n*-3 LC-PUFAs. Additionally, in an environment rich in *n*-3 LC-PUFAs, fish may preferentially obtain these compounds from the environment rather than from biosynthesis. However, their physiological demand for *n*-3 LC-PUFAs might be higher, potentially making them less resistant to a deficiency of dietary *n*-3 LC-PUFAs. On the contrary, in an environment where *n*-3 LC-PUFAs are not abundant, fish may have developed a stronger capacity for *n*-3 LC-PUFA synthesis and lower physiological requirements for these compounds, making fish less susceptible to a deficiency of dietary *n*-3 LC-PUFAs. The analysis of the fatty acid compositions of wild fish reveals significant differences in the *n*-3 LC-PUFA profiles among species with different habitats (saltwater and freshwater) and feeding habits (herbivorous, omnivorous, and carnivorous) [6]. In addition to growth performance, piscine biosynthetic capacity and physiological requirements of *n*-3 LC-PUFAs also affect the levels of retained *n*-3 LC-PUFAs in the fillet, which are associated with human acquisition of dietary *n*-3 LC-PUFAs.

Despite the benefits of fish oil for both farmed fish and consumers being largely attributed to *n*-3 LC-PUFAs, existing meta-analyses on the impact of replacing fish oil with plant oil in aquafeeds have predominantly focused on comparing various types of vegetable oils rather than using *n*-3 LC-PUFAs as the primary variable. In contrast, this study aimed to fill this gap by using a Bayesian meta-analysis to comprehensively analyze the effect of dietary *n*-3 LC-PUFA content on the growth performance and *n*-3 LC-PUFA composition among cultured fish with different feeding habits and habitats.

## 2. Materials and Methods

### 2.1. Study Search and Selection Criteria

**Figure 1:**
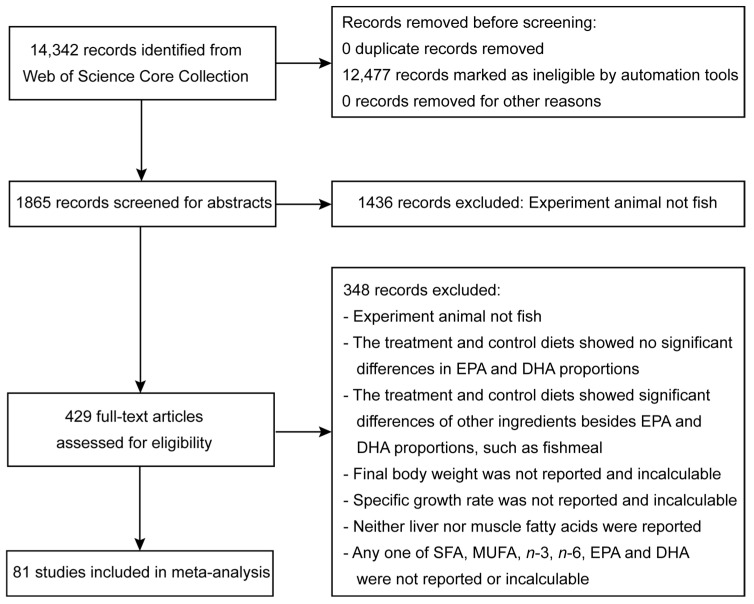
Flow diagram for study search and selection according to Preferred Reporting Items for Systematic Reviews and Meta-Analyses (PRISMA) [7].

The meta-analysis followed the guidelines of the PRISMA (the Preferred Reporting Items for Systematic Reviews and Meta-Analyses) 2020 statement [7]. The literature screening process is illustrated in Figure 1. To investigate the impact of plant or animal lipid substitutes for fish oil in aquafeeds on fish growth and fatty acid composition, this study first used “dietary fish oil” as a keyword to search the titles, keywords, and abstracts of articles in the Web of Science Core Collection database. Subsequently, the retrieved literature was preliminarily filtered using the filtering function of this database with the following criteria: Publication Years (2010–2023); Document Type (article); Language (English); Research Areas (Fisheries, Nutrition Dietetics, Marine Freshwater Biology, Biochemistry, Molecular Biology, Food Science Technology, Veterinary Sciences, Agriculture, Science Technology, Other Topics, Immunology, Chemistry, Physiology, Zoology, Endocrinology, Metabolism, Cardiovascular System, Cardiology, Cell Biology, Pharmacology Pharmacy, Environmental Sciences, Ecology, Biotechnology, Applied Microbiology, Toxicology, Life Sciences Biomedicine Other Topics, Reproductive Biology, Biodiversity, Conservation, Hematology, Oceanography, Developmental Biology, Evolutionary Biology, and Pathology). The selection of the Research Area, while aiming for maximum inclusiveness, excluded non-biological domains. The above search and filtering were completed on 24 May 2023, and the titles and abstracts of the filtered literature were downloaded for further manual screening.

During the manual screening process, we first excluded the literature that did not focus on fish as the research subject. Subsequently, we downloaded the full text of the retained literature. Through the full text, we further excluded articles based on the following criteria: (1) studies that did not consider the fatty acid composition of feed as a research variable; (2) studies where there was no significant differences in *n*-3 LC-PUFAs between the feeds used (e.g., studies using oxidized fish oil as a research variable); (3) studies where variables in the feed included factors other than fatty acid composition; (4) studies that did not report or where it was not possible to deduce the proportion of *n*-3 LC-PUFAs in the feed, the final body weight (FBW), specific growth rate (SGR), or the proportion of *n*-3 LC-PUFAs in the liver or muscle; and (5) articles not including the sample size, mean, and standard deviation (or standard error) of the dependent variable, such as studies presenting a pooled standard deviation.

### 2.2. Data Extraction and Analysis

The data extracted in this study included the scientific names of fish, fatty acid composition profiles in feed, liver, and muscle (percentages of saturated fatty acids (SFAs), monounsaturated fatty acids (MUFAs), PUFAs, *n*-3, *n*-6, EPA, and DHA), FBW (grams), and SGR. For the extracted data, apart from the feed fatty acid composition profile data, which consists of means, the data for other variables are composed of the mean, standard deviation (or standard error), and sample size. For the studies reporting absolute values of fatty acid content, we transformed them into the relative percentage of content using the formula “(absolute content of the fatty acid/total absolute content of fatty acids) multiplied by 100”. If the reported absolute content was expressed as g per 100 g of fatty acids, we directly used the value from the study as the relative percentage of content. For studies that reported standard errors, the standard error was converted to the standard deviation using the formula “standard deviation = standard error multiplied by square root of sample size” to be subsequently used in data analysis.

The present study aimed to investigate the differences in the FBW, SGR, hepatic, and muscle *n*-3 LC-PUFA depositions among fish with different feeding habits and habitats when exposed to various levels of *n*-3 LC-PUFAs in the diet. To this end, the fish were categorized into three distinct groups based on their trophic levels according to Daniel Pauly’s recommendations (https://fishbase.mnhn.fr/manual/FishBaseThe_ECOLOGY_Table.htm (accessed on 13 December 2023)): herbivorous fish (2.0–2.19), omnivorous fish (2.2–2.79), and carnivorous fish (>2.8). In the case of hybrid fish, their trophic level was determined by averaging the trophic levels of their parent species. Regarding habitats, we referred to the reported salinity levels of water in the literature and information from the FishBase database. This allowed us to categorize the study subjects into freshwater fish and marine fish. For the classification of diadromous fish species, we followed the salinity of water as reported in the literature. The above-mentioned information regarding the literature involved in this study is listed in Table A1. For data analysis, dietary categories rather than specific trophic levels were considered because trophic levels, which are determined by the species and external conditions such as prey, may not necessarily reflect the biological differences between fish.

This study utilized the corrected unbiased estimate of the standardized mean difference (SMD) as the effect size for the meta-analysis, namely Hedges’ g [8], to investigate the effects of replacing dietary fish oil with different levels of plant oil on the FBW, SGR, and the fatty acid composition in fish muscle and liver. We designated the group with the highest EPA + DHA content in each research report as the control group, and the remaining groups as the experimental groups. Considering that the impact of different levels of fish oil substitution on the research variables may vary, we further divided the experiment into four replacement levels (RL) based on the difference in EPA + DHA content in the feed between the experimental and control groups, namely 0 < RL ≤ 25, 25 < RL ≤ 50, 50 < RL ≤ 75, and 75 < RL ≤ 100. A positive effect size indicates that EPA + DHA (or *n*-3 LC-PUFAs for narrative) in the feed tends to increase the variable levels (FBW, SGR, liver EPA, liver DHA, muscle EPA, and muscle DHA). Additionally, we combined the different levels of plant oil replacement groups with the control group separately, forming independent sets of observational outcomes within the same research report. The calculation of effect sizes was performed using the “esc” R package (version 0.5.1) [9]. To assess potential publication bias in the studies included in the present research, we employed the “meta” R package (version 6.5-0) to generate a funnel plot [10,11].

After calculating effect size, we employed a Bayesian hierarchical model to analyze the pooled effects. This choice was made due to the skewed distribution of the obtained effect size and the relatively small sample size in some groups. To enhance the reliability of results, groups with fewer than three studies were excluded from the calculation of the pooled effect size. We employed Markov Chain Monte Carlo methods to simulate the posterior distribution of the effect size with weak informative priors. We assumed a prior probability distribution for the effect sizes of the FBW and SGR following a normal distribution N~(0, 1), meaning that the 95% credible interval (CrI) for the true effect size is [−2, 2]. This size aligns with previous meta-analyses using frequentist statistics for reporting the results of the FBW and SGR [12,13]. Due to the fact that the composition of fatty acids in fish bodies may essentially reflect the fatty acid composition in the feed [14], we adopted a broader range of prior probability distribution for the analysis of the effect sizes of EPA and DHA in the liver and muscle, namely N~(0, 4). The distribution assumption covered the range reported in a previous meta-analysis study [12]. Additionally, we assumed a prior distribution for the between-study variance of the effect size following a Half-Cauchy distribution HC~(0, 0.5). Before calculating the pooled effect size, we assessed the convergence of the model by computing the potential scale reduction factor (R-hat) and conducting posterior predictive checks. These analyses were performed using the “brms” R package (version 2.20.4) [15] and the guidance manual for conducting a Bayesian random-effects meta-analysis with this package [16]. All analyses were performed using R Statistical Software (v4.3.1; R Core Team 2023, Vienna, Austria). The R code and relevant parameters used in this article can be found in File S1. 

## 3. Results and Discussion

Unlike previous meta-analyses using a frequentist statistical approach [12,13], this study employed a Bayesian meta-analysis to investigate the effects of different levels of *n*-3 LC-PUFA intake on fish growth and *n*-3 LC-PUFA deposition in the liver and muscle. The effect sizes in the reports covered in the present study exhibited a skewed distribution. The use of Bayesian statistics can overcome the limitations imposed by the frequentist statistical approach on data distribution. Therefore, the Bayesian statistical methods employed in this study offered an alternative interpretation of the research outcomes on this specific topic.

### 3.1. Distribution of the Studies

**Figure 2:**
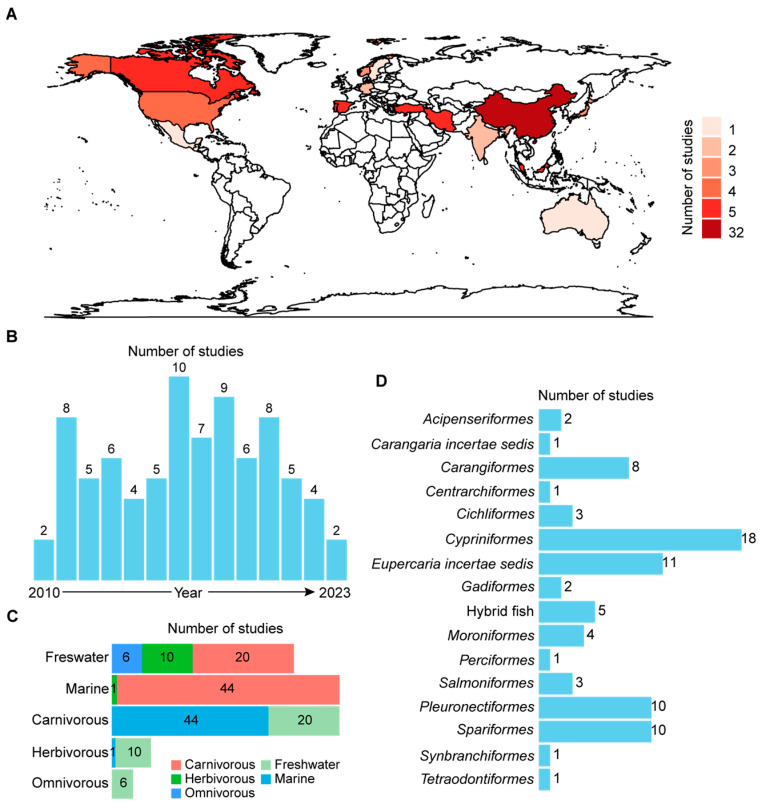
Distribution of studies included in this article based on country, publication date, and the feeding habits, habitats, and taxonomic orders of the studied fish. (**A**) Cumulative number of publications included in this article across different countries. (**B**) Cumulative number of publications included in this article from 2010 to 2023. (**C**) Cumulative number of publications related to the fish species studied in this article, categorized by habitat and feeding habit. (**D**) Cumulative number of publications related to the fish species studied in this article, categorized by taxonomic classification at the order rank.

After standardized screening, the present study included a total of 81 eligible research reports, serving as the subjects for the meta-analysis. These studies come from 17 countries and are distributed across all continents except Africa and Antarctica (Figure 2A). Among them, studies from China are the most numerous, accounting for 39.5%. This is not surprising, considering that in 2018, China’s aquaculture production represented 56.7% of the world’s total production [4]. Excluding China’s contribution, according to the World Bank’s 2023 data, the cumulative number of studies from high-income countries in these research papers is 61.2%, those from middle-income countries account for 24.5%, and studies from low-income countries contribute 14.3%. This result suggests that the substitution of fish oil with plant oils in aquafeeds is an important issue in the current aquaculture industry. The distribution of Publication Years in the research reports covered in this article is relatively uniform, from 2010 to 2023 (Figure 2B). The literature collection for this article concluded in May 2023, which may result in fewer publications for the year 2023.

This study aimed to analyze and compare the impact of dietary *n*-3 LC-PUFA levels on marine, freshwater, carnivorous, omnivorous, and herbivorous fish. A previous quantitative review study showed that muscle EPA and DHA compositions correlated with the trophic level, salinity, dietary lipid level (EPA only), temperature, and final body weight (DHA only) [14]. Furthermore, the analysis suggested no significant correlation between muscle EPA and DHA compositions and feeding duration [14]. Additionally, a recent meta-regression analysis found no significant correlation between growth and feeding duration concerning the replacement of dietary fish oil with various plant oils in rainbow trout, Atlantic salmon, tilapia, and gilthead sea bream [17]. Evidence shows a negative correlation between water temperature and the amount of *n*-3 LC-PUFAs in fish [14]. However, we did not include water temperature in this study, because it represents a more complex situation. The specific optimal growth temperature may not be coincidental with the water temperature reported in the studies included in this article. This is because the former reflects the inherent biological characteristics of the fish, while the latter may not affect the results of analyses that have control groups. Thus, since water temperature is closely related to the depth of water, it may not be proper to simply use the latitudinal distribution of fish (i.e., tropical fish, temperate fish, and polar fish) to differentiate the optimal growth temperature of fish. Additionally, the final body weights of fish depend not only on water temperature but also varies with different initial body weights and rearing durations [18].

In Figure 2C, we present the distribution of the number of studies under these five categories. The data collected in this article are derived from 43 fish species (including 3 hybrid fish), comprising 20 freshwater fish and 23 marine fish. The number of reports on freshwater fish is less than that of marine fish, accounting for 44% and 56%, respectively. This result may be associated with the higher demand of dietary fish oil for the production of most marine fish compared to freshwater fish aquafeeds [2], attracting more research attention, despite the fact that the world’s aquaculture production of freshwater fish is six times higher than that of marine fish [4]. Among freshwater fish, the reported percentages for herbivorous, omnivorous, and carnivorous fish were 28%, 17%, and 56%, respectively. In marine fish, these percentages were 2%, 0%, and 98%, respectively. On the other hand, among carnivorous fish, freshwater fish and marine fish accounted for 31% and 69%, respectively, while this ratio was 91% and 9% for herbivorous fish. The only herbivorous marine fish species investigated in this study was *Siganus canaliculatus* [19]. There were no marine omnivorous fish species found in the collected research reports. Fish species with different habitats and diets showed a significant disparity in the quantity of research reports. This may indicate diverse research attention on the topic of fish oil substitution with plant oil among the five mentioned categories of fish. Taxonomic distribution at the order rank of fish species included in this study is shown in Figure 2D. Considering the number of species, *Cypriniformes* was the most represented order, followed by *Eupercaria incertae sedis*, *Salmoniformes*, and *Spariformes*.

### 3.2. Composition of Dietary Fatty Acids

**Figure 3:**
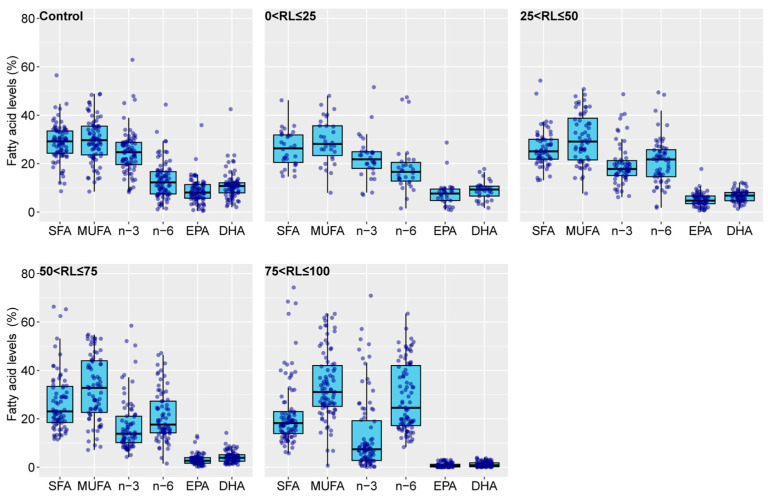
Percentages of various fatty acids in the feeds used in the research reports covered in this study. The control group refers to the group in a specific study with the highest *n*-3 LC-PUFA content in the feed. 0 < RL ≤ 25, 25 < RL ≤ 50, 50 < RL ≤ 75, and 75 < RL ≤ 100 denote the percentages of *n*-3 LC-PUFAs in the feed of a particular group in a study relative to its highest content group, falling within the ranges of 0–25%, 25–50%, 50–75%, and 75–100%, respectively. SFA, saturated fatty acid; MUFA, monounsaturated fatty acid; *n*-3, *n*-3 polyunsaturated fatty acids; *n*-6, *n*-6 polyunsaturated fatty acids; EPA, eicosapentaenoic acid; DHA, docosahexaenoic acid; *n*-3 LC-PUFA, *n*-3 long-chain polyunsaturated fatty acid; RL, replacement level.

In the research reports covered in this article, the overall fatty acid percentages of EPA and DHA in feeds were skewed. Therefore, we categorized the different feed treatments in each study into control and experimental groups. The control group comprised the group with the highest percentage of *n*-3 LC-PUFAs (typically using fish oil as the fat source). The experimental groups were further divided based on the percentage of *n*-3 LC-PUFAs relative to the control group into four intervals: 0–25%, 25–50%, 50–75%, and 75–100%, denoted as 0 < RL ≤ 25, 25 < RL ≤ 50, 50 < RL ≤ 75, and 75 < RL ≤ 100, respectively. We collected data and, where necessary, calculated the percentages of SFAs, MUFAs, *n*-3, *n*-6, as well as EPA and DHA relative to the total fatty acids for the five aforementioned groups (Figure 3). With the increasing replacement of fish oil with vegetable oil, the proportion of EPA and DHA in feeds and the differences in this proportion among studies are gradually decreasing. Fishmeal is a frequent feed component that contains a certain amount of fish oil. Therefore, utilizing the percentage of dietary EPA + DHA rather than the percentage of dietary vegetable oil replacing fish oil prevents the omission of residual fish oil in fishmeal from compromising the outcomes of this study. The average distribution of SFAs and MUFAs in each group ranged from 22.22% to 28.75% and from 33.77% to 29.35%, respectively, with small variations (*n* = 30–87; SD of 2.38 and 1.95, respectively). The average percentages of *n*-3 PUFAs, EPA, and DHA in each group ranged from 13.23% to 24.65%, 0.84% to 8.63%, and 1.11% to 10.83%, respectively. As the replacement level increased, *n*-3 PUFAs, EPA, and DHA showed a gradual decrease. In contrast, the average percentage of *n*-6 PUFAs in each group showed a gradual increment with the increased replacement level, ranging from 13.07% to 28.92%. Furthermore, based on the average values of EPA and DHA in each group, the proportions of EPA + DHA to the control group were 85.77% (0 < RL ≤ 25), 60.48% (25 < RL ≤ 50), 37.98% (50 < RL ≤ 75), and 10.02% (75 < RL ≤ 100) of the values found in the control group. From Figure 3, it can be concluded that the control group of some research reports showed a proportion of EPA close to zero. These studies administered DHA alone instead of fish oil [20]. The differences between *n*-3 PUFAs and *n*-6 PUFAs among groups are consistent with the fact that plant oils generally have lower levels of *n*-3 PUFAs and a higher content of *n*-6 PUFAs. Furthermore, the absence of EPA and DHA in plant oils allows for the content of *n*-3 LC-PUFAs in feeds to directly reflect the level of fish oil substitution by vegetable oil.

### 3.3. Model Convergence and Publication Bias

The R-hat values for the models used to calculate pooled effect sizes in the present study ranged between 0.9998 and 1.0009 (Table S1). Posterior predictive checks revealed that the density distribution of the posterior data (y_rep_) closely matched the density distribution of the calculated effect size (y) (Figures S1–S6). Thus, the models used in the present study were deemed suitable for calculating the pooled effect sizes for the FBW, SGR, liver, and muscle EPA and DHA [21].

Publication bias occurs when studies with statistically significant results are more likely to be published than those with non-significant results, leading to an asymmetrical distribution of study outcomes. When there is no publication bias, small studies usually have large standard errors, and effect sizes with large standard errors have wider confidence intervals, resulting in a scatter plot with the effect size on the horizontal axis and the standard error of the effect size on the vertical axis, exhibiting an upside-down funnel [22]. The points at the top of this plot are relatively concentrated, while the points at the bottom are more dispersed. 

As shown in the funnel plot in (Figures S7–S12), the scatter points for FBW and SGR roughly exhibited an inverted triangular distribution, suggesting a relatively low likelihood of publication bias. Although the scatter points in the funnel plot for liver and muscle EPA and DHA are mostly located to the right of the zero-effect line, there is still a notable concentration of data points at the top, with a more dispersed pattern below. This asymmetrical distribution of scatter points for liver and muscle EPA and DHA can be attributed to the direct reflection of the differences in dietary EPA and DHA, which determine the EPA and DHA proportions in fish bodies.

### 3.4. Growth Performance

#### 3.4.1. FBW

**Figure 4:**
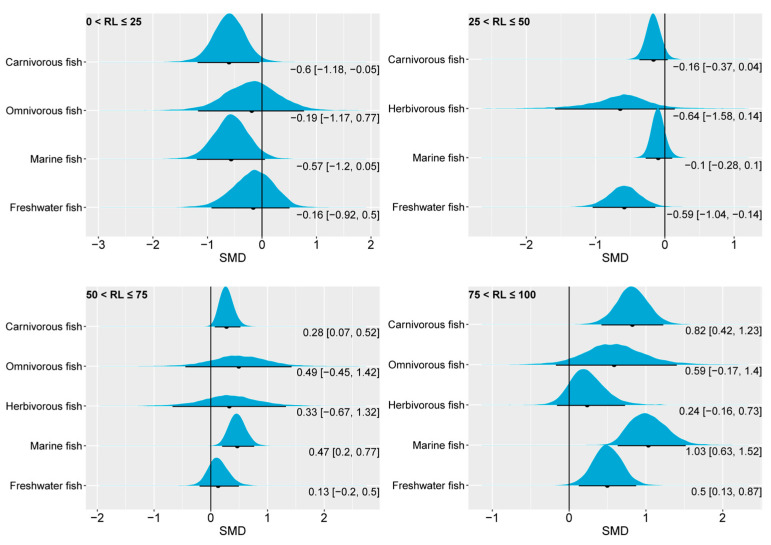
The impact of dietary *n*-3 LC-PUFA levels on the FBWs of carnivorous, omnivorous, herbivorous, marine, and freshwater fish. The blue curve represents the posterior distribution of the SMD for the pooled effect size of the FBW. The black dot and black line below each blue curve indicate the median and 95% CrI of the posterior distribution, respectively. These values are also numerically displayed to the bottom right of each blue curve. The numerical values to the left of the brackets represent medians, and the values inside the brackets represent the 95% CrI. An SMD < 0 indicates that the increase in the FBW is attributed to lower dietary *n*-3 LC-PUFAs, while an SMD > 0 indicates that the increase in FBW is attributed to higher dietary *n*-3 LC-PUFAs. In general, an interval within the brackets containing 0 suggests that changes in dietary *n*-3 LC-PUFAs have no significant effect on FBW, while not containing 0 indicates a significant effect. RL, replacement level; FBW, final body weight; SMD, standardized mean difference; CrI, credible interval; *n*-3 LC-PUFAs, *n*-3 long-chain polyunsaturated fatty acids.

Our investigation explored the effects of varying levels of *n*-3 LC-PUFAs in fish feed on the FBW. In scenarios where the *n*-3 LC-PUFA content was reduced by 0–25% compared to the control group (which had 19.46% of total fatty acids, as shown in Figure 3), the 95% CrI of the posterior SMD for omnivorous, marine, and freshwater fish encompassed the numerical value 0 (Figure 4). This suggests that a slight reduction in the *n*-3 LC-PUFA content had a limited impact on the FBW, with carnivorous fish even exhibiting a positive effect, as indicated by a 95% CrI falling below 0. From the total of 24 studies analyzed, 10 reported effect-size values within [0.006, 1.379], while the remaining 14 studies had a range within [−0.3061, −8.9756]. Marine fish dominated this classification with 20 studies, while 4 studies focused on freshwater fish, aligning with the distribution characteristics observed in pooled effect-size intervals. As the replacement level of *n*-3 LC-PUFAs in the experimental group reached the range of 25 < RL ≤ 50 (with a mean of 11.77% of total fatty acids, Figure 3), no significant change was observed in the pooled effect sizes for carnivorous, herbivorous, and marine fish compared to the control. However, the 95% CrI of the posterior SMD of freshwater fish is clearly located to the left of 0, indicating that reducing *n*-3 LC-PUFAs by 25–50% of the control benefits the FBW performance of freshwater fish. When the replacement level increased to 50 < RL ≤ 75, the posterior distributions of the SMD indicated no significant differences in the pooled effect sizes of the FBW for herbivorous, omnivorous, and freshwater fish compared to the control. Yet, the 95% CrI of the posterior SMD of the FBW for carnivorous fish and marine fish was distributed on the right side of 0, suggesting a significant decrease in the FBW for these groups when the *n*-3 LC-PUFA proportion was reduced to 50–75% (mean of 7.39 %, Figure 3) of the control group. In the case of further increases in the replacement level to 75 < RL ≤ 100, the pooled effect sizes of the FBW in herbivorous and omnivorous fish showed no significant differences compared to the control, as indicated by their 95% CrI of the posterior SMD around 0. The 95% CrI of the posterior SMD of carnivorous fish, marine fish, and freshwater fish exhibited a positive range in all cases, indicating that reducing the dietary *n*-3 LC-PUFAs to 75–100% of the control group is detrimental to the FBW performances of these species. Overall, from the perspective of FBW, our analysis results revealed that marine fish and carnivorous fish have a high demand for dietary *n*-3 LC-PUFAs, followed by freshwater fish. The FBWs of omnivorous and herbivorous fish, on the other hand, were less responsive to changes in *n*-3 LC-PUFAs in feed.

#### 3.4.2. SGR

**Figure 5:**
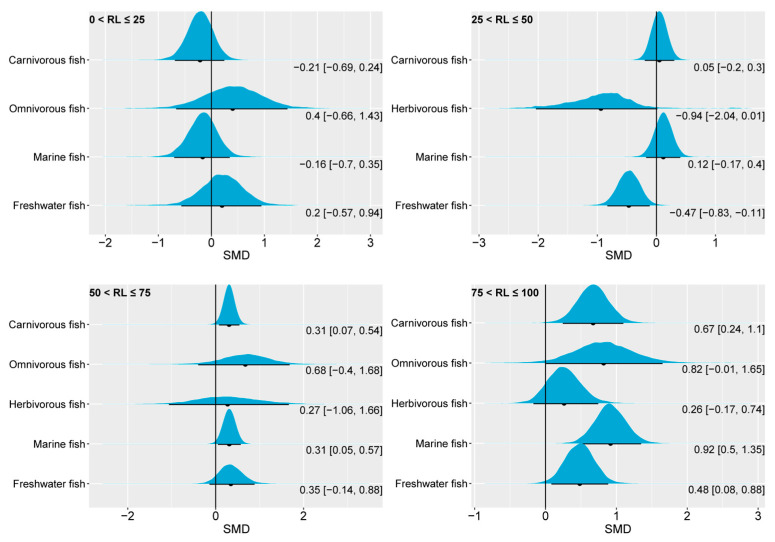
The impact of dietary *n*-3 LC-PUFA levels on the SGRs of carnivorous, omnivorous, herbivorous, marine, and freshwater fish. The blue curve represents the posterior distribution of the SMD for the pooled effect size of the SGR. The black dot and black line below each blue curve indicate the median and 95% CrI of the posterior distribution, respectively. These values are also numerically displayed to the bottom right of each blue curve. The numerical values to the left of the brackets represent medians, and the values inside the brackets represent the 95% CrI. An SMD < 0 indicates that the increase in SGR is attributed to lower dietary *n*-3 LC-PUFAs, while an SMD > 0 indicates that the increase in SGR is attributed to higher dietary *n*-3 LC-PUFAs. In general, an interval within the brackets containing 0 suggests that changes in dietary *n*-3 LC-PUFAs have no significant effect on SGR, while not containing 0 indicates a significant effect. RL, replacement level; SGR, specific growth rate; SMD, standardized mean difference; CrI, credible interval; *n*-3 LC-PUFAs, *n*-3 long-chain polyunsaturated fatty acids.

The pooled effect size of the SGR in carnivorous, omnivorous, herbivorous, marine, and freshwater fish showed similarities with the FBW at different oil substitution levels (Figure 5). This similarity is not surprising, as studies that involve the substitution of dietary fish oil with plant oil are typically conducted over the same durations and with similar initial weights. Indeed, the SGR depends on the number of days, as well as the initial and final weights. Briefly, regarding the SGR values, carnivorous fish and marine fish did not show significant differences compared to the control when the dietary *n*-3 LC-PUFA replacement level was less than 50% of the value in the control group. However, when the level exceeded 50% of the control group, the SGR performances of carnivorous fish and marine fish were suboptimal. Freshwater fish, on the other hand, only exhibited decreased SGR values when the replacement level surpassed 75% of the control group. In the present study, herbivorous and omnivorous fish showed the insensitivity of the SGR to changes in the content of dietary *n*-3 LC-PUFAs.

Our findings are consistent with previous studies on growth performance using frequency-based meta-analyses. For instance, the meta-analysis performed by Sales and Glencross indicates that substituting 50–100% of fish oil with various plant oils (including canola oil, linseed oil, and soybean oil) also has an unfavorable impact on fish growth [12]. In addition, the substitution of 100% fish oil with plant oil also had a significant negative effect on the growths of both freshwater and marine fish. As in the present study, the impact on marine fish was more pronounced than in freshwater fish [12]. Similarly, another meta-analysis utilizing a frequency-based statistical approach revealed a significantly negative pooled effect size of substituting fish oil with plant oil on the SGR of marine and carnivorous fish [13]. In this regard, there is evidence indicating that dietary plant oil can trigger growth hormone resistance in marine fish [23]. Additionally, the role of dietary *n*-3 LC-PUFAs in enhancing immune function may collectively contribute to the impact of dietary *n*-3 LC-PUFA levels on the growth performances of marine and carnivorous fish [24].

### 3.5. n-3 LC-PUFA Composition

#### 3.5.1. Liver EPA and DHA

The impact of changes in the ratio of dietary *n*-3 LC-PUFAs on the hepatic content of EPA was generally consistent in different fish species, with higher levels of dietary *n*-3 LC-PUFAs often resulting in higher liver EPA proportions. This observation is not surprising, as numerous studies indicate that the fatty acid composition in fish body fat broadly reflects the proportion of fatty acids ingested [14].

**Figure 6:**
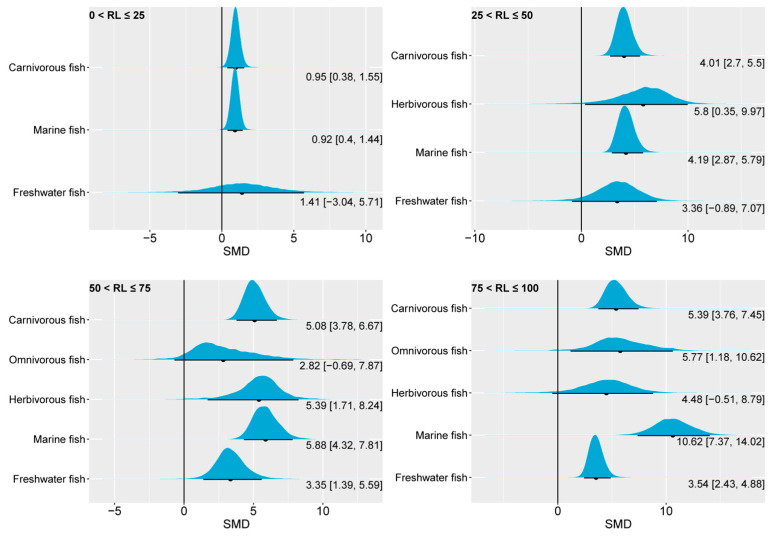
The impact of dietary *n*-3 LC-PUFA levels on liver EPA of carnivorous, omnivorous, herbivorous, marine, and freshwater fish. The blue curve represents the posterior distribution of the SMD for the pooled effect size of liver EPA. The black dot and black line below each blue curve indicate the median and 95% CrI of the posterior distribution, respectively. These values are also numerically displayed to the bottom right of each blue curve. The numerical values to the left of the brackets represent medians, and the values inside the brackets represent the 95% CrI. An SMD < 0 indicates that the increase in liver EPA is attributed to lower dietary *n*-3 LC-PUFAs, while an SMD > 0 indicates that the increase in liver EPA is attributed to higher dietary *n*-3 LC-PUFAs. In general, an interval within the brackets containing 0 suggests that changes in dietary *n*-3 LC-PUFAs have no significant effect on liver EPA, while not containing 0 indicates a significant effect. RL, replacement level; EPA, eicosapentaenoic acid; SMD, standardized mean difference; CrI, credible interval; *n*-3 LC-PUFAs, *n*-3 long-chain polyunsaturated fatty acids.

Nevertheless, we observed differences in the liver EPA content of fish with different feeding habits and habitats at varying substitution levels of *n*-3 LC-PUFAs in the feed (Figure 6). In carnivorous and marine fish, reducing dietary *n*-3 LC-PUFAs to 0–25% of the control significantly decreased the liver EPA content. This effect was evidenced by the 95% CrI of the posterior distributions of the SMD of liver EPA, which consistently exceeded 0.

A comparison between the two groups, 0 < RL ≤ 25 and 25 < RL ≤ 50, showed significant differences in the 95% CrI for carnivorous fish and marine fish. However, between the groups 25 < RL ≤ 50 and 50 < RL ≤ 75, there was evident overlap in the 95% CrI for carnivorous fish and marine fish. This suggests that liver EPA remained relatively stable when the oil substitution level increased from 25–50% to 50–75%. Nevertheless, increasing the substitution level to 75–100% in carnivorous fish resulted in only a slight increase in the 95% CrI, while marine fish exhibited a more pronounced increase. This may be attributed to the fact that the data for carnivorous fish consist of 31% freshwater fish and 69% marine fish, and the liver EPA content of freshwater fish seems less sensitive to changes in the dietary *n*-3 LC-PUFA content. Indeed, Figure 6 shows that the 95% CrI of liver EPA of freshwater fish contained 0 at substitution levels from 0 to 50%. When the substitution level increased from 50–75% to 75–100%, the median of the SMD of freshwater fish only increased from 3.35 to 3.54.

**Figure 7:**
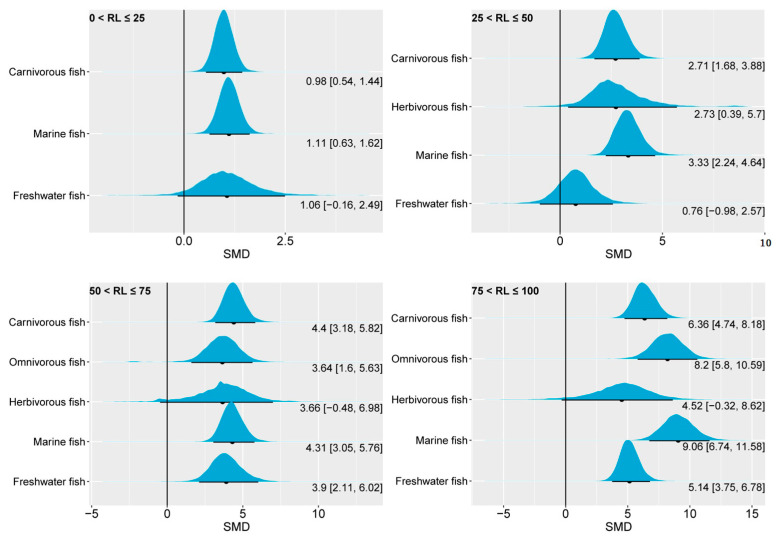
The impact of dietary *n*-3 LC-PUFA levels on liver DHA of carnivorous, omnivorous, herbivorous, marine, and freshwater fish. The blue curve represents the posterior distribution of the SMD for the pooled effect size of liver DHA. The black dot and black line below each blue curve indicate the median and 95% CrI of the posterior distribution, respectively. These values are also numerically displayed to the bottom right of each blue curve. The numerical values to the left of the brackets represent medians, and the values inside the brackets represent the 95% CrI. An SMD < 0 indicates that the increase in liver DHA is attributed to lower dietary *n*-3 LC-PUFAs, while an SMD > 0 indicates that the increase in liver DHA is attributed to higher dietary *n*-3 LC-PUFAs. In general, an interval within the brackets containing 0 suggests that changes in dietary *n*-3 LC-PUFAs have no significant effect on liver DHA, while not containing 0 indicates a significant effect. RL, replacement level; DHA, docosahexaenoic acid; SMD, standardized mean difference; CrI, credible interval; *n*-3 LC-PUFAs, *n*-3 long-chain polyunsaturated fatty acids.

The response of liver DHA to oil substitution and varying dietary *n*-3 LC-PUFA levels was similar to that of liver EPA, wherein a noticeable decrease in the liver DHA content was observed when aquafeeds contained reduced levels of *n*-3 LC-PUFAs (Figure 7). When the substitution level was between 0 and 50%, there was no significant difference in liver DHA content compared to the control, but when the oil substitution level was between 50 and 100%, the 95% CrI of liver DHA of freshwater fish showed a clear above-zero range, and this was more pronounced compared to the 95% CrI of liver EPA. As mentioned before, for carnivorous fish and marine fish, there was a significant overlap in 95% CrI of liver EPA between the 25–50% and 50–75% oil substitution levels, whereas this phenomenon was less pronounced in the 95% CrI of liver DHA. This suggests that liver DHA, compared to EPA, was more dependent on changes in dietary *n*-3 LC-PUFAs.

#### 3.5.2. Muscle EPA and DHA

**Figure 8:**
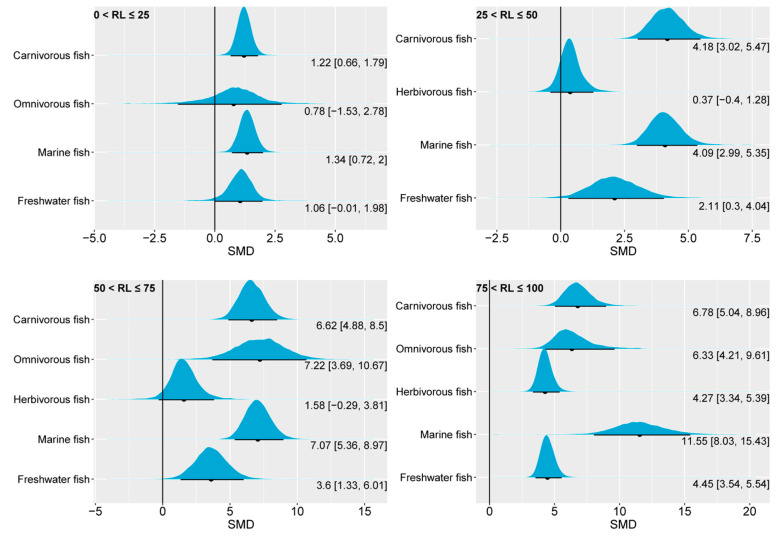
The impact of dietary *n*-3 LC-PUFA levels on muscle EPA of carnivorous, omnivorous, herbivorous, marine, and freshwater fish. The blue curve represents the posterior distribution of the SMD for the pooled effect size of muscle EPA. The black dot and black line below each blue curve indicate the median and 95% CrI of the posterior distribution, respectively. These values are also numerically displayed to the bottom right of each blue curve. The numerical values to the left of the brackets represent medians, and the values inside the brackets represent the 95% CrI. An SMD < 0 indicates that the increase in muscle EPA is attributed to lower dietary *n*-3 LC-PUFAs, while an SMD > 0 indicates that the increase in muscle EPA is attributed to higher dietary *n*-3 LC-PUFAs. In general, an interval within the brackets containing 0 suggests that changes in dietary *n*-3 LC-PUFAs have no significant effect on muscle EPA, while not containing 0 indicates a significant effect. RL, replacement level; EPA, eicosapentaenoic acid; SMD, standardized mean difference; CrI, credible interval; *n*-3 LC-PUFAs, *n*-3 long-chain polyunsaturated fatty acids.

**Figure 9:**
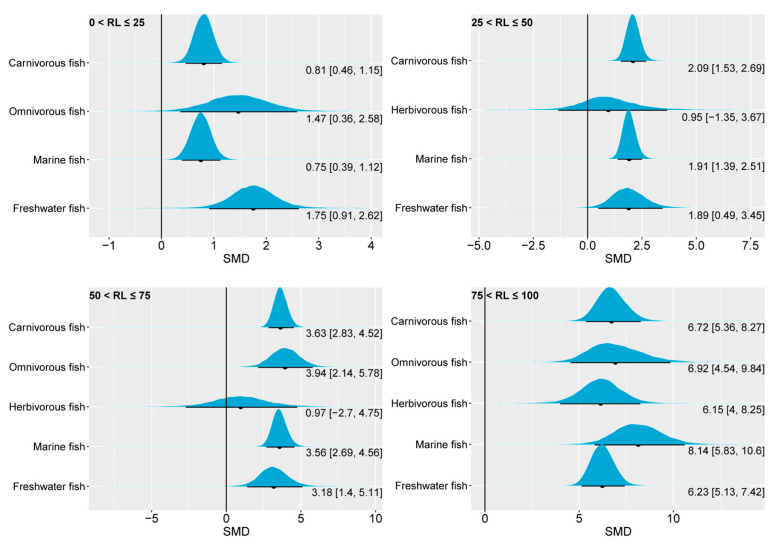
The impact of dietary *n*-3 LC-PUFA levels on muscle DHA of carnivorous, omnivorous, herbivorous, marine, and freshwater fish. The blue curve represents the posterior distribution of the SMD for the pooled effect size of muscle DHA. The black dot and black line below each blue curve indicate the median and 95% CrI of the posterior distribution, respectively. These values are also numerically displayed to the bottom right of each blue curve. The numerical values to the left of the brackets represent medians, and the values inside the brackets represent the 95% CrI. An SMD < 0 indicates that the increase in muscle DHA is attributed to lower dietary *n*-3 LC-PUFAs, while an SMD > 0 indicates that the increase in muscle DHA is attributed to higher dietary *n*-3 LC-PUFAs. In general, an interval within the brackets containing 0 suggests that changes in dietary *n*-3 LC-PUFAs have no significant effect on muscle DHA, while not containing 0 indicates a significant effect. RL, replacement level; DHA, docosahexaenoic acid; SMD, standardized mean difference; CrI, credible interval; *n*-3 LC-PUFAs, *n*-3 long-chain polyunsaturated fatty acids.

In general, the performance of EPA and DHA in the fish muscle was similar to that in the liver in response to changes in dietary *n*-3 LC-PUFAs (Figure 8 and Figure 9). However, the response of muscle EPA and DHA to changes in dietary *n*-3 LC-PUFAs appeared to be more sensitive than in the liver. This is consistent with the fact that the skeletal muscle primarily plays a role in retaining EPA and DHA. Nevertheless, the proportions of muscle EPA and DHA exhibited different responses to changes in dietary *n*-3 LC-PUFAs depending on the fish category.

Regarding muscle EPA, carnivorous and herbivorous fish exhibited remarkable differences in response to changes in dietary *n*-3 LC-PUFAs (Figure 8). Indeed, in all four groups of this study, the 95% CrI of the SMD for muscle EPA in carnivorous fish was significantly greater than 0, indicating that increasing the amount of *n*-3 LC-PUFAs in the feed can effectively enhance the levels of muscle EPA in this type of fish. On the other hand, the 95% CrI of herbivorous fish only demonstrated an above-zero range in the highest oil substitution-level group. The insensitivity of muscle EPA content in herbivorous fish to changes in dietary *n*-3 LC-PUFAs may be essentially related to their ability to synthesize EPA [2]. Interestingly, in the 50 < RL ≤ 75 and 75 < RL ≤ 100 groups, the 95% CrI for omnivorous fish was significantly higher than for herbivorous fish and comparable to that of carnivorous fish. This may imply a limited EPA synthesis capacity in omnivorous fish. Similar to carnivorous fish, the muscle EPA content of marine fish exhibited a dependence on dietary *n*-3 LC-PUFAs across the four replacement level groups, an effect possibly linked to the absence of (or weak capability in) the expression of genes related to EPA synthesis in marine fish [2]. The range of the 95% CrI of freshwater fish only showed a zero-included range in the 0–25% oil substitution group. Nonetheless, the response of muscle EPA content in freshwater fish to higher levels of *n*-3 LC-PUFA substitution may primarily originate from the data of carnivorous and omnivorous fish within it.

Compared to muscle EPA, muscle DHA showed a higher sensitivity to changes in dietary *n*-3 LC-PUFA levels (Figure 9). Except for herbivorous fish, where the 95% CrI of the SMD of muscle DHA only exhibited an above-zero range in the highest oil substitution-level group, the muscle DHA of other fish species in the four groups considered in this study all showed an above-zero range for the 95% CrI. Additionally, except for marine fish, which showed a slightly higher 95% CrI, the 95% CrI of muscle DHA remained similar across fish species as the substitution level increased. Since the biosynthesis of DHA is downstream of EPA, the similarity in the effect of dietary *n*-3 LC-PUFA levels on the muscle DHA content of different fish species may indicate that their biosynthetic capacities for DHA may be comparable. Since some lower aquatic organisms have the ability to synthesize *n*-3 LC-PUFAs [3], the dietary requirements of fish for DHA are likely to meet primarily through the trophic chain.

### 3.6. Insights into Dietary n-3 LC-PUFA Levels Affecting Growth of Marine and Carnivorous Fish

The findings of the present study demonstrate that both the growth performance and accumulation of *n*-3 LC-PUFAs in marine and carnivorous fish are highly sensitive to the dietary content of *n*-3 LC-PUFAs. Thus, gaining a deeper understanding of the intermediary metabolic processes involving *n*-3 LC-PUFAs in marine and carnivorous fish is of significance for improving their aquaculture production and quality.

Unlike terrestrial animals, fish, particularly carnivorous fish, tend to utilize amino acids rather than carbohydrates to supply energy needs. The protein requirement for carnivorous fish is typically around 46% [25]. Amino acid usage for energy metabolism in fish is consistent with their weak carbohydrate digestion and breakdown capabilities [26]. Previously, we reported that on the first day of refeeding after 19-day fasting, the recovery of liver glycogen of gilthead sea bream (*Sparus aurata*) fed high-protein diets was significantly faster than that in fish fed high-carbohydrate diets [27]. This may reflect the efficient breakdown of amino acids in carnivorous fish such as *S. aurata*. Moreover, some studies found that fish also tend to utilize amino acid breakdown energy rather than carbohydrates for lipid synthesis [28,29].

Although carnivorous fish preferably use amino acids to obtain energy, studies demonstrated the potential of carnivorous fish to utilize dietary carbohydrates for growth. Extensive studies on *S. aurata* showed that disrupting the amino acid energy pathway can increase the activity of rate-limiting enzymes in the glycolytic pathway while inhibiting the activities of key enzymes in gluconeogenesis. Nevertheless, glutamine and glutamate represent the primary ATP sources from amino acids in fish [30]. Indeed, supplementation with dietary glutamine suppressed the activities of enzymes related to glycolysis, such as glucokinase, 6-phosphofructo-1-kinase, and pyruvate kinase in the liver [31]. On the other hand, in the *S. aurata* liver, glutamate dehydrogenase silencing decreased the glutamate, glutamine, and aspartate aminotransferase activities while increasing the 6-phosphofructo-1-kinase/fructose-1,6-bisphosphatase activity ratio, suggesting an activation of glycolytic flux [32]. Similarly, both the inhibition and knockdown of liver alanine aminotransferase also tended to increase the activities of key enzymes in glycolysis [33,34]. Sterol regulatory element-binding protein 1a (SREBP1a), a potent transcription factor with a major role in the control of lipid biosynthesis, also participates in the regulation of carbohydrate metabolism-related enzymes in carnivorous fish [35,36]. Recently, we utilized chitosan–tripolyphosphate nanoparticles complexed with a plasmid expressing the N-terminus transactivating domain of SREBP1a to overexpress this transcriptional factor in the liver of *S. aurata*. The hepatic expression of SREBP1a caused a multigenic effect, leading to increased lipid biosynthesis from dietary carbohydrates [37,38].

Modulating the proportion of *n*-3 LC-PUFAs in feed may be one approach to enhancing the ability of fish, particularly marine and carnivorous species, to utilize dietary carbohydrates. High-carbohydrate or high-fat diets are prone to trigger metabolic dysregulation, and the relatively weak glucose-utilization ability in fish may exacerbate this progression. Studies on mammals have shown that *n*-3 LC-PUFAs help improve the glucose metabolism of subjects with insulin resistance, while such benefits for healthy individuals are limited [39,40]. In teleosts, dietary supplementation with *n*-3 LC-PUFAs exerts varying effects on glucose metabolism. For instance, differences in glycogen deposition have been noted in the hepatocytes of European seabass fed fish oil-rich diets compared to land animal fat-based diets [41]. However, the impact of *n*-3 LC-PUFAs on resting-state circulatory glucose levels in carnivorous fish appears to be minimal [42]. Conversely, substituting dietary fish oil with vegetable oil was shown to influence serum postprandial glucose levels in *S. aurata* [43], suggesting a nuanced effect of *n*-3 LC-PUFAs on fish glucose metabolism. Additionally, the inclusion of linseed oil, which is rich in α-linolenic acid, has been linked to the reduced activity of glucose-6-phosphate dehydrogenase in the liver of *S. aurata* [44], further indicating the potential for dietary *n*-3 LC-PUFAs to modulate basal glucose metabolic processes in fish.

Therefore, the *n*-3 LC-PUFA-mediated enhancement of fish growth performance, especially in marine and carnivorous species, may due to the improvement of insulin sensitivity and the consequent utilization of dietary carbohydrates [45,46]. Our recent report also show that *n*-3 LC-PUFAs can improve the growth performances of marine and carnivorous fish [47], which could be due to improved insulin sensitivity. However, the impact of dietary *n*-3 LC-PUFA content on growth performance is not always significant and may interact with other factors such as temperature, dietary lipid content, and salinity. For instance, dietary *n*-3 LC-PUFAs may not benefit the growth of carnivorous fish at certain replacement levels, and higher water temperatures might enhance the contribution of saturated fatty acids to growth [48].

## 4. Conclusions

The present study was the first to employ Bayesian statistical methods and conduct a meta-analysis using *n*-3 LC-PUFA levels in aquafeeds as the primary variable to analyze their impact on *n*-3 LC-PUFA content and growth in cultured fish. The methodology herein used avoided the impact of residual oil in fishmeal, which is approximately 10% *w*/*w* and may be an overlooked factor when studying the fish oil requirements of farmed fish [49]. The results of this study can contribute to the optimization of fish oil levels in aquafeeds and promote the development of more sustainable aquaculture practices. Our findings showed that the growth performances of freshwater and herbivorous fish were less affected by low levels of *n*-3 LC-PUFAs in the feed. In contrast, for optimal growth of marine fish, dietary *n*-3 LC-PUFA levels should be at least approximately 7% of the total fatty acids (mean value of the 50 < RL ≤ 75 group). In terms of nutritional value, and with the exception of herbivorous fish, changes in the proportion of dietary *n*-3 LC-PUFAs were directly reflected in the muscle *n*-3 LC-PUFA content of cultured fish. Further promising research efforts include incorporating *n*-3 LC-PUFA-rich algae and bacterial oil into the diet [50,51,52] and developing transient gene therapy methods to express exogenous enzymes that enable *n*-3 LC-PUFA biosynthesis in culture fish without producing genetically modified organisms [47].

## Data Availability

The data supporting the conclusions of this study will be made available from the authors upon request.

## Supplementary Materials

The following supporting information can be downloaded at: https://www.mdpi.com/article/10.3390/ani14142118/s1, Figure S1: Posterior predictive check of simulated FBW from the posterior predictive distribution. Figure S2: Posterior predictive check of simulated SGR from the posterior predictive distribution. Figure S3: Posterior predictive check of simulated liver EPA from the posterior predictive distribution. Figure S4: Posterior predictive check of simulated liver DHA from the posterior predictive distribution. Figure S5: Posterior predictive check of simulated muscle EPA from the posterior predictive distribution. Figure S6: Posterior predictive check of simulated muscle DHA from the posterior predictive distribution. Figure S7: Funnel plot of FBW. Figure S8: Funnel plot of SGR. Figure S9: Funnel plot of liver EPA. Figure S10: Funnel plot of liver DHA. Figure S11: Funnel plot of muscle EPA. Figure S12: Funnel plot of muscle DHA. Table S1: The R-hat values of b_Intercept for the models used to calculate the pooled effect sizes of the groups in the present study. File S1: R code for calculating the effect sizes of each study and R code for Bayesian posterior probability simulation.

## Appendix A

**Table A1:** The literature used for data analysis in this article.

Scientific Name	Habitat	Feeding Habits	Oil Sources	Replacement Level	Year	Ref.
*Acanthopagrus schlegelii*	M	C	Cc, Pr, Su, Ar, ED	75, 100	2017	[53]
*Acanthopagrus schlegelii*	M	C	Ar	50, 75, 100	2017	[54]
*Acanthopagrus schlegelii*	M	C	Fi-So, So	25, 50, 75	2019	[55]
*Acanthopagrus schlegelii*	M	C	Cr	25, 50	2013	[56]
*Argyrosomus regius*	M	C	Li-Pa-Ra, Fi-Li-Pa-Ra	25, 50, 75	2019	[57]
*Argyrosomus regius*	M	C	Li-Ra	25, 50, 75, 100	2023	[58]
*Argyrosomus regius*	M	C	Fi-So, So	25, 50, 75, 100	2016	[59]
*Barbonymus gonionotus*	F	O	Cr, Li	100	2018	[60]
*Barbonymus gonionotus*	F	O	Li, Fi-Li	25, 75, 100	2017	[61]
*Barbonymus gonionotus*	F	O	Cr, Li, Cr + Li	100	2020	[62]
*Brachymystax lenok*	F	C	Fi-Li, Li	25, 50, 75, 100	2019	[63]
*Carassius gibelio*	F	O	Pk, Ra	100	2016	[64]
*Ctenopharyngodon idellus*	F	H	Pk	50, 75, 100	2011	[65]
*Ctenopharyngodon idellus*	F	H	Pk	100	2015	[66]
*Ctenopharyngodon idellus*	F	H	So	75	2022	[67]
*Ctenopharyngodon idellus*	F	H	Ov, Pe, Li	100	2018	[68]
*Cyclopterus lumpus*	M	C	Fi-Ra, Ra	50, 75, 100	2021	[69]
*Cyprinus carpio*	F	C	Ra	100	2015	[70]
*Dicentrarchus labrax*	M	C	So	100	2016	[71]
*Dicentrarchus labrax*	M	C	Fi-Pt-Ma, Pt-Ma	50, 75, 100	2018	[41]
*Dicentrarchus labrax*	M	C	Fi-Ra-Pa	75	2021	[46]
*Dicentrarchus labrax*	M	C	Fi-Ra, Ra	50, 100	2016	[72]
*Epinephelus fuscoguttatus* x *E. lanceolatus*	M	C	W, Fi-W	25, 50, 75	2019	[73]
*Epinephelus fuscoguttatus* x *E. lanceolatus*	M	C	Cr, Su, T, Ov, Ri, Fi-Cr-Su-T-Ov-Ri	75, 100	2020	[74]
*Gadus morhua*	M	C	Cm	100	2014	[75]
*Gadus morhua*	M	C	Cm, Fi-Cm	25	2013	[76]
*Huso huso*	F	C	Su, So, Ra, Su-So-Ra	50, 100	2011	[77]
*Huso huso*	F	C	So, Ra	100	2010	[78]
*Huso huso* x *Acipenser ruthenus*	F	C	Li, So	100	2018	[79]
*Labeo rohita*	F	O	G, Fi-G	25, 50, 75, 100	2022	[80]
*Larimichthys crocea*	M	C	Pn	25, 50, 75, 100	2020	[81]
*Larimichthys crocea*	M	C	So, B	75	2012	[82]
*Larimichthys crocea*	M	C	So, Li, Ra, Pe	50	2017	[83]
*Megalobrama amblycephala*	F	C	So, Pk	100	2016	[84]
*Megalobrama amblycephala*	F	C	Ra, Pa, Pe, So	100	2015	[85]
*Megalobrama amblycephala*	F	C	So, DHA + So	50, 75, 100	2020	[86]
*Megalobrama amblycephala*	F	C	So, Pa, Fi-So-Pa	75, 100	2017	[87]
*Micropterus salmoides*	F	C	Cc, Ra, Li	75	2022	[88]
*Monopterus albus*	F	C	Li, Pk, Pe, So, Ar-Pe	100	2011	[89]
*Morone chrysops* x *M. saxatilis*	M	C	Cr	75	2011	[90]
*Mylopharyngodon piceus*	F	C	Ra, Fi-Ra	25, 50, 75, 100	2011	[91]
*Nibea albiflora*	M	C	Fi-Li-Pk	75	2020	[92]
*Nibea coibor*	M	C	Pa, Fi-Pa	25, 50, 75, 100	2016	[93]
*Oncorhynchus mykiss*	F	C	Fi-Ra-Li-Pa-Su	25, 50, 75	2018	[94]
*Oncorhynchus mykiss*	F	C	Fl, Fi-Fl	50, 75	2013	[95]
*Oncorhynchus mykiss*	F	C	Cm, Fi-Cm	50, 75	2014	[96]
*Oncorhynchus mykiss*	F	C	Fi-La	25, 50	2017	[97]
*Oncorhynchus mykiss*	F	C	Se, Su, Li	75	2015	[98]
*Onychostoma macrolepis*	F	O	So, Li, Al, So-Li-Al	25, 75, 100	2021	[99]
*Oreochromis niloticus*	F	H	Li	50, 75	2016	[100]
*Oreochromis niloticus*	F	H	Fi-Pa, Pa	50, 75, 100	2018	[101]
*Oreochromis niloticus* var. GIFT	F	H	Cm, Fi-Cm	25, 75, 100	2020	[102]
*Oreochromis* sp.	F	H	Pr, Ra, Su, Pa	100	2016	[103]
*Polydactylus sexfilis*	M	C	Fi-So	25, 50, 75	2014	[104]
*Rachycentron canadum*	M	C	So	25, 50	2011	[105]
*Rachycentron canadum*	M	C	So	50, 75	2013	[106]
*Rachycentron canadum*	M	C	Pr, Su	50, 100	2018	[107]
*Salmo salar*	M	C	Ra	75	2022	[108]
*Salmo salar*	M	C	Fi-Li-Pa-Ra	50	2011	[109]
*Salmo salar*	F	C	Ra, Mi-Ra	25	2023	[20]
*Salvelinus alpinus*	F	C	Fi-Ra-Pa, Ra-Pa	75	2010	[110]
*Scatophagus argus*	F	C	So	100	2021	[111]
*Seriola dumerili*	M	C	Fi-Li-Pa, Li-Pa	50, 75	2018	[112]
*Seriola lalandi*	M	C	Ra, Pt	100	2012	[113]
*Seriola lalandi*	M	C	Ra-Pa	50	2020	[114]
*Seriola quinqueradiata*	M	C	Ra, Fi-Ra	25, 50, 75	2017	[115]
*Siganus canaliculatus*	M	H	Fi-So, So	50, 75	2012	[19]
*Solea senegalensis*	M	C	Li, So	50, 75	2013	[116]
*Solea senegalensis*	M	C	Fi-Ra-So-Li, Ra-So-Li	25, 50, 75	2014	[117]
*Solea senegalensis*	M	C	Fi-So-Ra-Li, So-Ra-Li	50, 100	2019	[118]
*Sparidentex hasta*	M	C	Fi-Ra, Fi-Su, Fi-B, Ra, Su, B	50, 75	2016	[119]
*Sparidentex hasta*	M	C	Oc	75, 100	2015	[120]
*Sparus aurata*	M	C	Fi-Li-Ra-Pa	75	2019	[121]
*Sparus aurata*	M	C	Cm, Ch, Fi-Cm, Fi-Ch	50, 75	2020	[122]
*Sparus aurata*	M	C	Ra + Li + Pa	50	2016	[123]
*Sparus aurata*	M	C	So	25, 50	2011	[124]
*Takifugu rubripes*	M	C	So, Li	25, 50, 75, 100	2011	[125]
*Tor tambroides*	F	H	Fi-Ov, Ov	100	2012	[126]
*Tor tambroides*	F	H	Cr, Li, Cr + Li	100	2021	[127]
*Totoaba macdonaldi*	M	C	B	75, 100	2018	[128]
*Trachinotus ovatus*	M	C	Fi-So-Ra-Pr	50	2020	[129]

For each of the 81 articles included in this study, the table shows the fish’s scientific name, habitat (F, freshwater; M, marine), feeding habits (C, carnivorous; H, herbivorous; O, omnivorous), oil sources (Al, algae; Ar, arachidonic acid-enriched oil; B, beef tallow; Cm, *Camelina* seed oil; Ch, chia oil; Cc, coconut oil; Cr, corn oil; ED, EPA + DHA-enriched oil; Fi, fish oil; Fl, flaxseed oil; G, groundnut oil; La, laurel oil; Li, linseed oil; Ma, mammalian fat; Mi, microbial oil; Oc, oleic acid; Ov, olive oil; Pa, palm oil; Pn, palmitin; Pe, peanut oil; Pr, *Perilla* oil; Pk, pork lard; Pt, poultry oil; Ra, rapeseed oil; Ri, rice oil; Se, sesame oil; So, soybean oil; Su, sunflower oil; T, tea oil; W, wheat germ oil), percentage of replacement level (25: 0–25; 50: 25–50; 75: 50–75; 100: 75–100), year of publication, and reference.
