# Supplementary material for: Bayesian Meta-Analysis: Impacts of Eating Habits and Habitats on Omega-3 Long-Chain Polyunsaturated Fatty Acid Composition and Growth in Cultured Fish
Source: Animals (Basel). 2024 Jul 20;14(14):2118. doi: 10.3390/ani14142118 (PMC11273610; doi:10.3390/ani14142118)
Supplement: Supplementary file 1 [file animals-14-02118-s001.zip › animals-3103784-supplementary.pdf]

Supplementary Materials

Table S1. The R-hat values of b_Intercept for the models used to calculate pooled effect sizes of groups in the present study.

Group	b_Intercept	Group	b_Intercept	Group	b_Intercept
MuscleEPAOmni75	1.000341	LiverEPAFresh75	1.000608	FBWFresh100	1.000783
SGRFresh75	1.000187	MuscleDHAOmni75	1.00103	LiverEPAMarine75	1.000764
MuscleDHAMarine25	1.0001	LiverDHAHerb75	1.000663	MuscleEPAFresh25	1.001078
LiverEPAMarine25	1.000826	FBWOmni75	1.00026	LiverEPACarn50	1.00083
LiverDHAMarine25	1.000278	FBWOmni100	0.999874	MuscleEPAHerb50	1.002266
MuscleEPAMarine50	1.000272	SGROmni25	1.001054	LiverDHACarn50	1.00154
LiverDHAOmni75	1.000122	SGROmni75	1.000346	LiverEPAHerb100	1.000705
LiverDHAMarine100	1.000672	LiverEPAFresh50	1.001522	LiverDHAFresh25	1.00037
SGRCarn75	1.000566	LiverDHAMarine75	1.001439	LiverDHACarn100	1.000163
MuscleDHAMarine50	1.000514	SGRFresh100	1.000472	MuscleDHACarn75	1.00044
SGRMarine100	1.000308	SGRHerb100	1.000175	FBWCarn50	1.000058
MuscleDHAOmni100	1.001305	SGRFresh50	0.999869	LiverDHACarn25	1.00064
LiverDHAOmni100	1.000057	LiverDHACarn75	1.000903	MuscleDHAFresh25	1.00064
SGRCarn50	1.000523	LiverEPACarn75	1.001123	LiverEPAMarine100	1.000134
FBWMarine50	1.00043	LiverEPAFresh25	1.000813	MuscleDHAMarine75	1.00016
FBWCarn25	1.000684	LiverDHAHerb50	1.001589	LiverEPAFresh100	1.000162
MuscleDHACarn25	1.000714	MuscleDHAMarine100	1.000529	MuscleEPAHerb75	1.001007
SGRMarine25	1.000481	MuscleEPACarn100	1.000243	MuscleDHAFresh50	1.001644
MuscleEPAFresh100	1.000127	FBWHerb75	1.000496	FBWMarine75	1.000352
MuscleDHACarn50	1.000784	FBWCarn100	1.000941	LiverEPAHerb50	1.000748
MuscleDHAFresh100	1.000094	LiverEPACarn25	1.000463	LiverEPACarn100	1.000535
MuscleEPACarn75	1.002506	FBWMarine100	1.000469	MuscleEPAFresh75	1.001423
LiverEPAOmni100	1.00255	SGRCarn25	1.000167	SGROmni100	1.000422
FBWFresh25	1.000485	FBWCarn75	1.000953	MuscleEPAOmni100	1.003302
MuscleEPAFresh50	1.001252	MuscleEPAHerb100	1.001088	MuscleEPAOmni25	1.002855
SGRMarine50	1.00059	LiverEPAMarine50	1.000414	LiverEPAOmni75	1.001658
MuscleDHAOmni25	1.000364	MuscleEPACarn50	1.000429	MuscleDHAHerb75	1.000927
FBWFresh75	1.000116	FBWFresh50	1.000117	LiverDHAMarine50	1.001005
SGRCarn100	1.00028	MuscleEPAMarine75	1.000521	LiverEPAHerb75	1.001618
FBWHerb100	1.000328	MuscleEPAMarine100	0.999972	SGRHerb75	1.001202
SGRMarine75	1.000068	LiverDHAFresh100	1.001932	FBWHerb50	1.003811
LiverDHAFresh50	1.000898	MuscleDHAFresh75	1.001117	MuscleDHAHerb50	1.002869
FBWOmni25	1.000398	LiverDHAFresh75	1.000643	SGRHerb50	1.003871
MuscleDHAHerb100	1.000214	MuscleEPACarn25	1.000311	LiverDHAHerb100	1.008641
MuscleEPAMarine25	1.00008	SGRFresh25	1.000266		

FBWMarine25	1.000798	MuscleDHACarn100	1.000703

FBW, final body weight; SGR, specific growth rate; Herb, herbivorous fish; Omni, omnivorous fish; Carn, carnivorous fish; Fresh, freshwater fish; Marine, marine fish; EPA, eicosapentaenoic acid; DHA, docosahexaenoic acid; 25, 0 < RL ≤ 25; 50, 25 < RL ≤ 50; 75, 50 < RL ≤ 75; 100, 75 < RL ≤ 100.

File S1

R code for calculating the effect sizes of each study:

```
library(esc)

data.effect_size <- esc::effect_sizes(data = Mydata, fun = "esc_mean_sd", grp1m = grp1m,
                      grp1n = grp1n, grp1sd = grp1sd, grp2m = grp2m,
                      grp2n = grp2n,    grp2sd = grp2sd, es.type = c("g"))
```

R code for Bayesian posterior probability simulation:

```
library(brms)

#First step: state the prior. Prior1 is applied for modeling FBW and SGR. Prior2 is applied for modeling EPA and DHA in the liver and muscle.

prior1 <- c(prior(normal(0,1), class = Intercept), prior(cauchy(0,0.5), class = sd))

prior2 <- c(prior(normal(0,4), class = Intercept), prior(cauchy(0,0.5), class = sd))


#Then, using Markov Chain Monte Carlo methods to simulate the posterior distribution of effect sizes

data.posterior <- brm(es|se(se) ~ 1 + (1|study), data = data.effect_size, prior = prior1, iter = 8000)

data.posterior <- brm(es|se(se) ~ 1 + (1|study), data = data.effect_size, prior = prior2, iter = 8000)
```

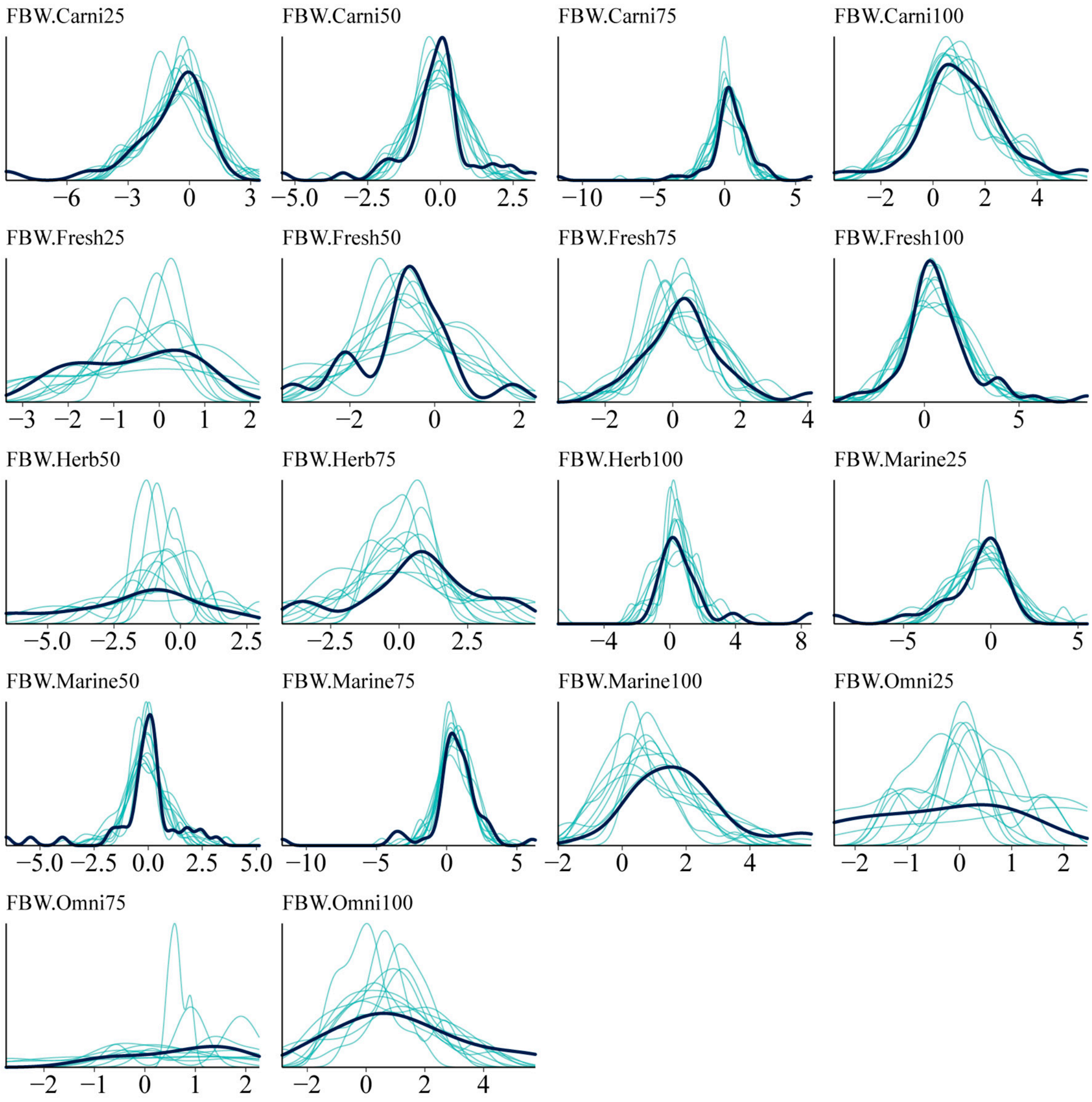

Figure S1. Posterior predictive check of simulated FBW from the posterior predictive distribution. The 10 light blue lines represent the density distribution data of 10 simulated FBW effect sizes randomly drawn from the posterior predictive distribution. The dark blue line represents the actual density distribution of FBW effect sizes encompassed in this study. If the assumed model is convergent and valid, the simulated density distribution should roughly resemble the actual distribution. FBW, final body weight; Herb, herbivorous fish; Omni, omnivorous fish; Carn, carnivorous fish; Fresh, freshwater fish; Marine, marine fish; 25, $0 < RL \leq 25$; 50, $25 < RL \leq 50$; 75, $50 < RL \leq 75$; 100, $75 < RL \leq 100$.

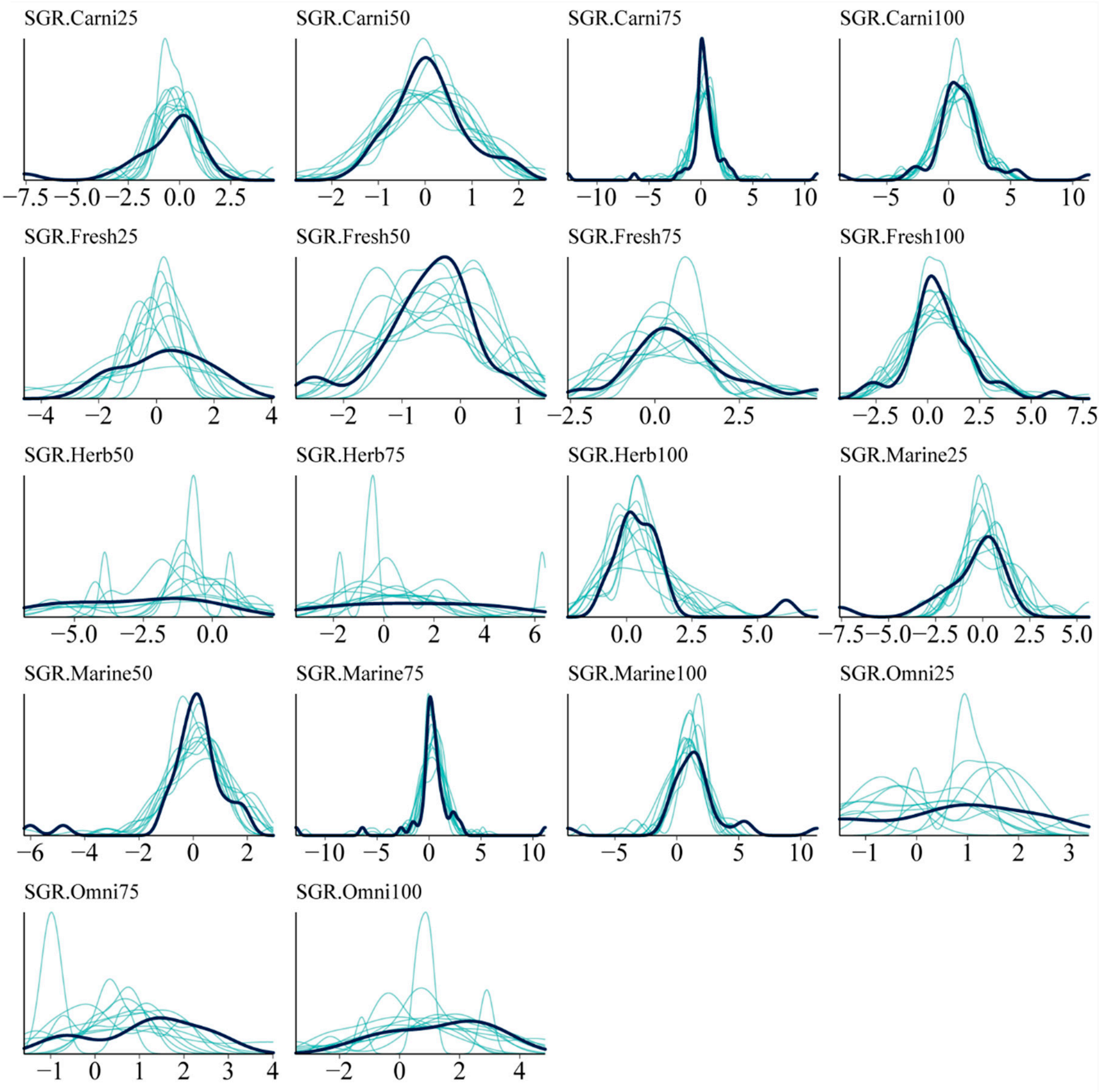

Figure S2. Posterior predictive check of simulated SGR from the posterior predictive distribution. The 10 light blue lines represent the density distribution data of 10 simulated SGR effect sizes randomly drawn from the posterior predictive distribution. The dark blue line represents the actual density distribution of SGR effect sizes encompassed in this study. If the assumed model is convergent and valid, the simulated density distribution should roughly resemble the actual distribution. SGR, specific growth rate; Herb, herbivorous fish; Omni, omnivorous fish; Carn, carnivorous fish; Fresh, freshwater fish; Marine, marine fish; 25, 0 < RL ≤ 25; 50, 25 < RL ≤ 50; 75, 50 < RL ≤ 75; 100, 75 < RL ≤ 100.

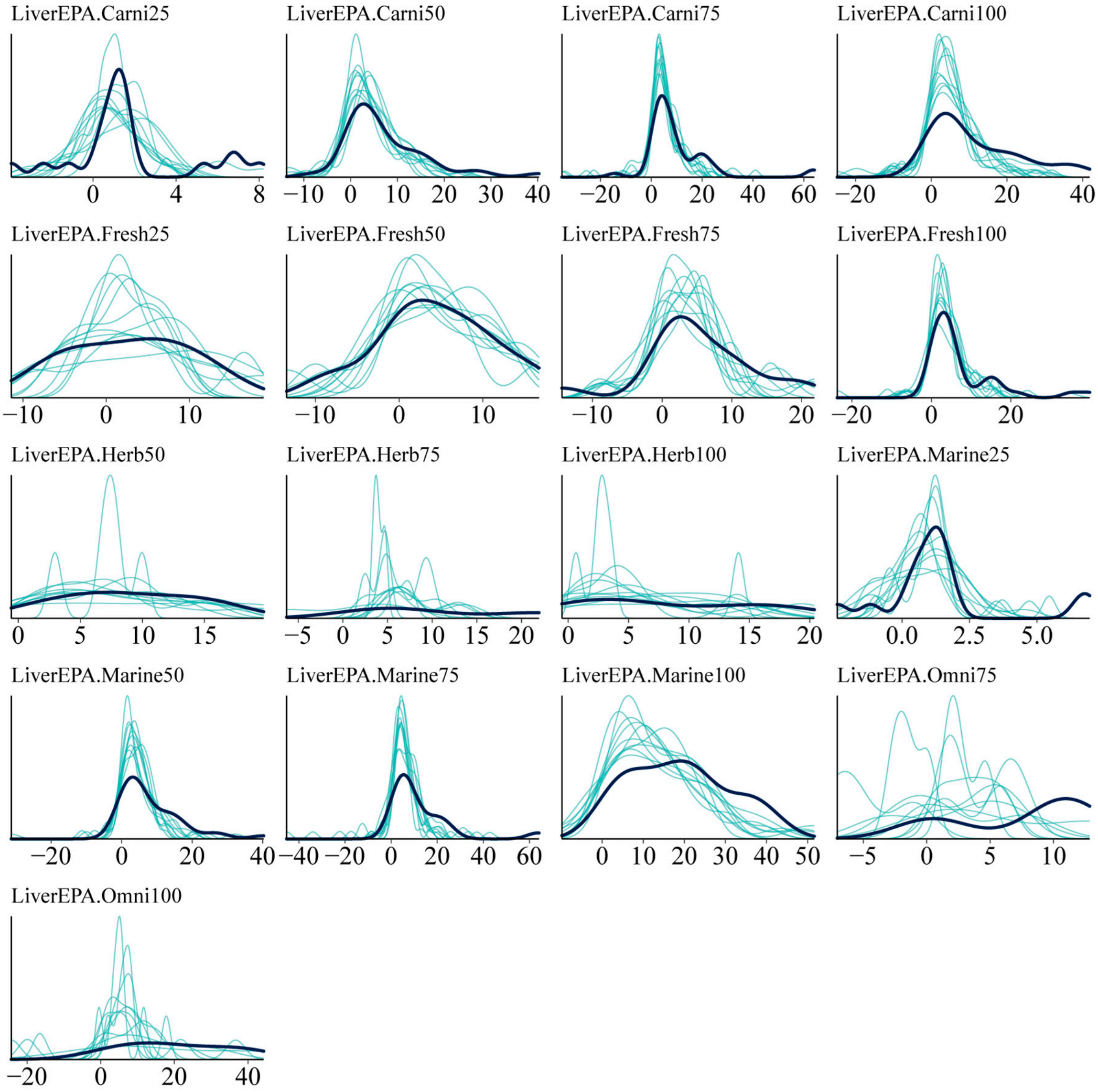

Figure S3. Posterior predictive check of simulated liver EPA from the posterior predictive distribution. The 10 light blue lines represent the density distribution data of 10 simulated liver EPA effect sizes randomly drawn from the posterior predictive distribution. The dark blue line represents the actual density distribution of liver EPA effect sizes encompassed in this study. If the assumed model is convergent and valid, the simulated density distribution should roughly resemble the actual distribution. Herb, herbivorous fish; Omni, omnivorous fish; Carn, carnivorous fish; Fresh, freshwater fish; Marine, marine fish; EPA, eicosapentaenoic acid; 25, 0 < RL ≤ 25; 50, 25 < RL ≤ 50; 75, 50 < RL ≤ 75; 100, 75 < RL ≤ 100.

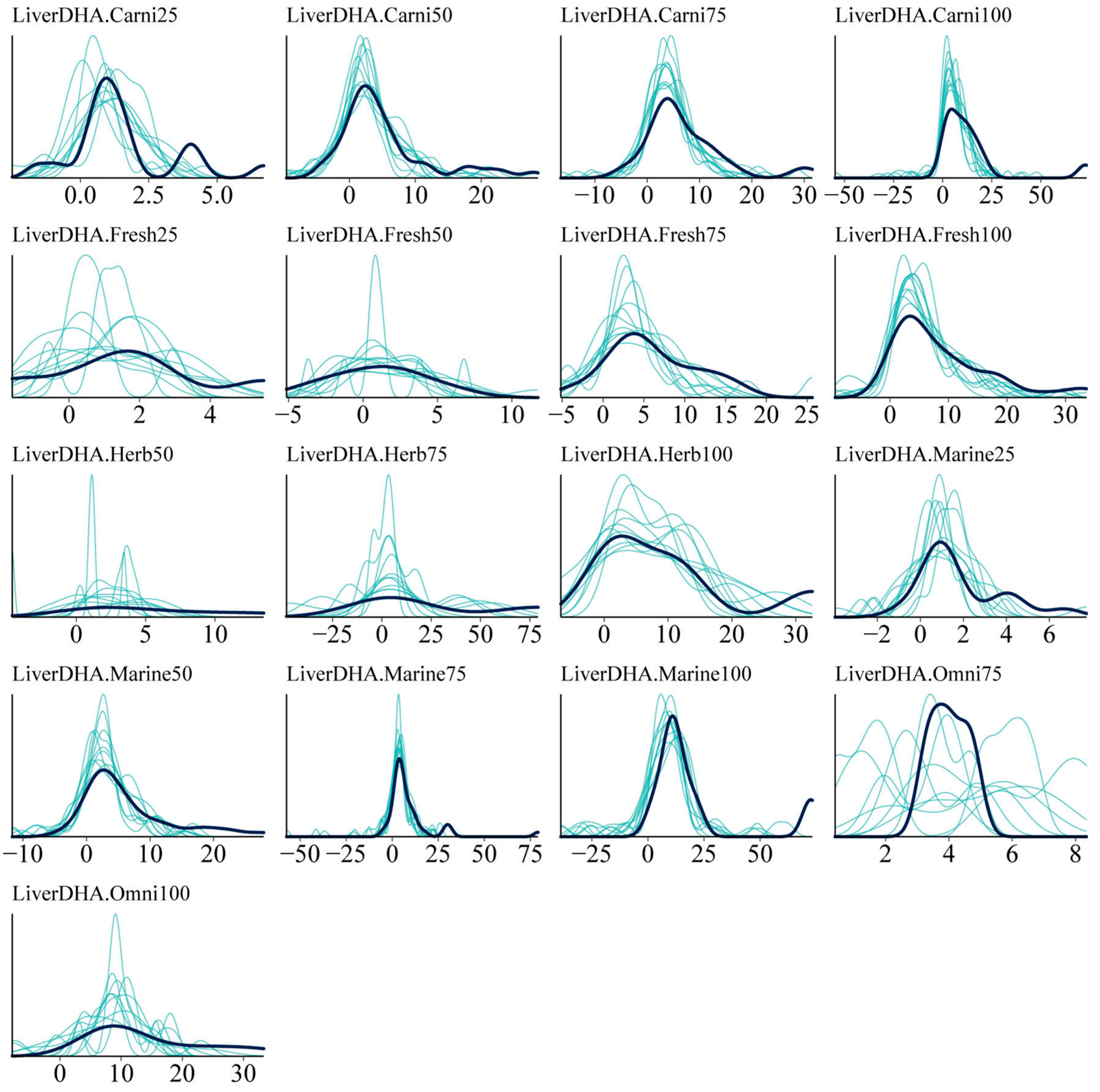

Figure S4. Posterior predictive check of simulated liver DHA from the posterior predictive distribution. The 10 light blue lines represent the density distribution data of 10 simulated liver DHA effect sizes randomly drawn from the posterior predictive distribution. The dark blue line represents the actual density distribution of liver DHA effect sizes encompassed in this study. If the assumed model is convergent and valid, the simulated density distribution should roughly resemble the actual distribution. Herb, herbivorous fish; Omni, omnivorous fish; Carn, carnivorous fish; Fresh, freshwater fish; Marine, marine fish; DHA, docosahexaenoic acid; 25, $0 < RL \leq 25$; 50, $25 < RL \leq 50$; 75, $50 < RL \leq 75$; 100, $75 < RL \leq 100$.

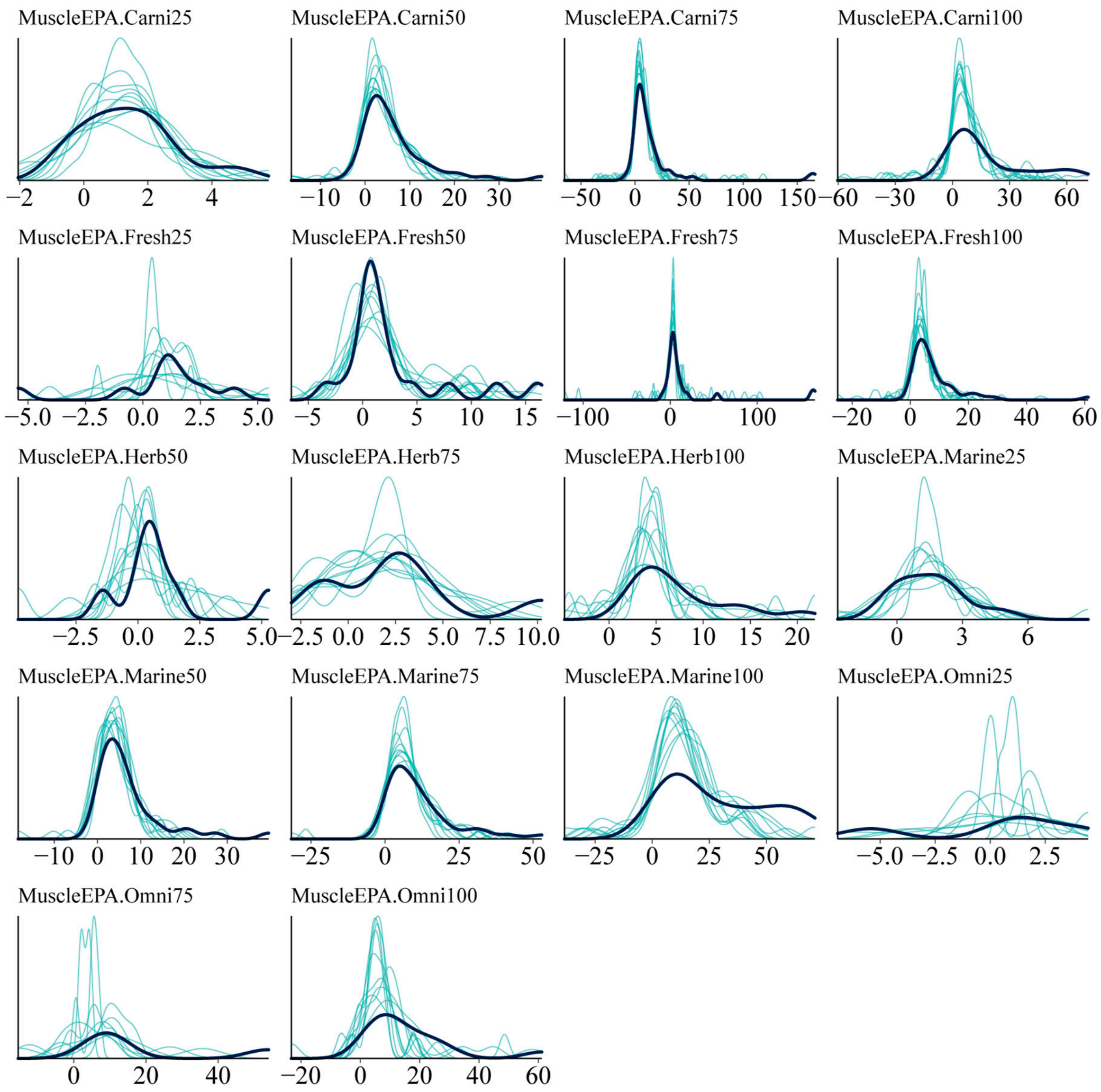

Figure S5. Posterior predictive check of simulated muscle EPA from the posterior predictive distribution. The 10 light blue lines represent the density distribution data of 10 simulated muscle EPA effect sizes randomly drawn from the posterior predictive distribution. The dark blue line represents the actual density distribution of muscle EPA effect sizes encompassed in this study. If the assumed model is convergent and valid, the simulated density distribution should roughly resemble the actual distribution. Herb, herbivorous fish; Omni, omnivorous fish; Carn, carnivorous fish; Fresh, freshwater fish; Marine, marine fish; EPA, eicosapentaenoic acid; 25, 0 < RL ≤ 25; 50, 25 < RL ≤ 50; 75, 50 < RL ≤ 75; 100, 75 < RL ≤ 100.

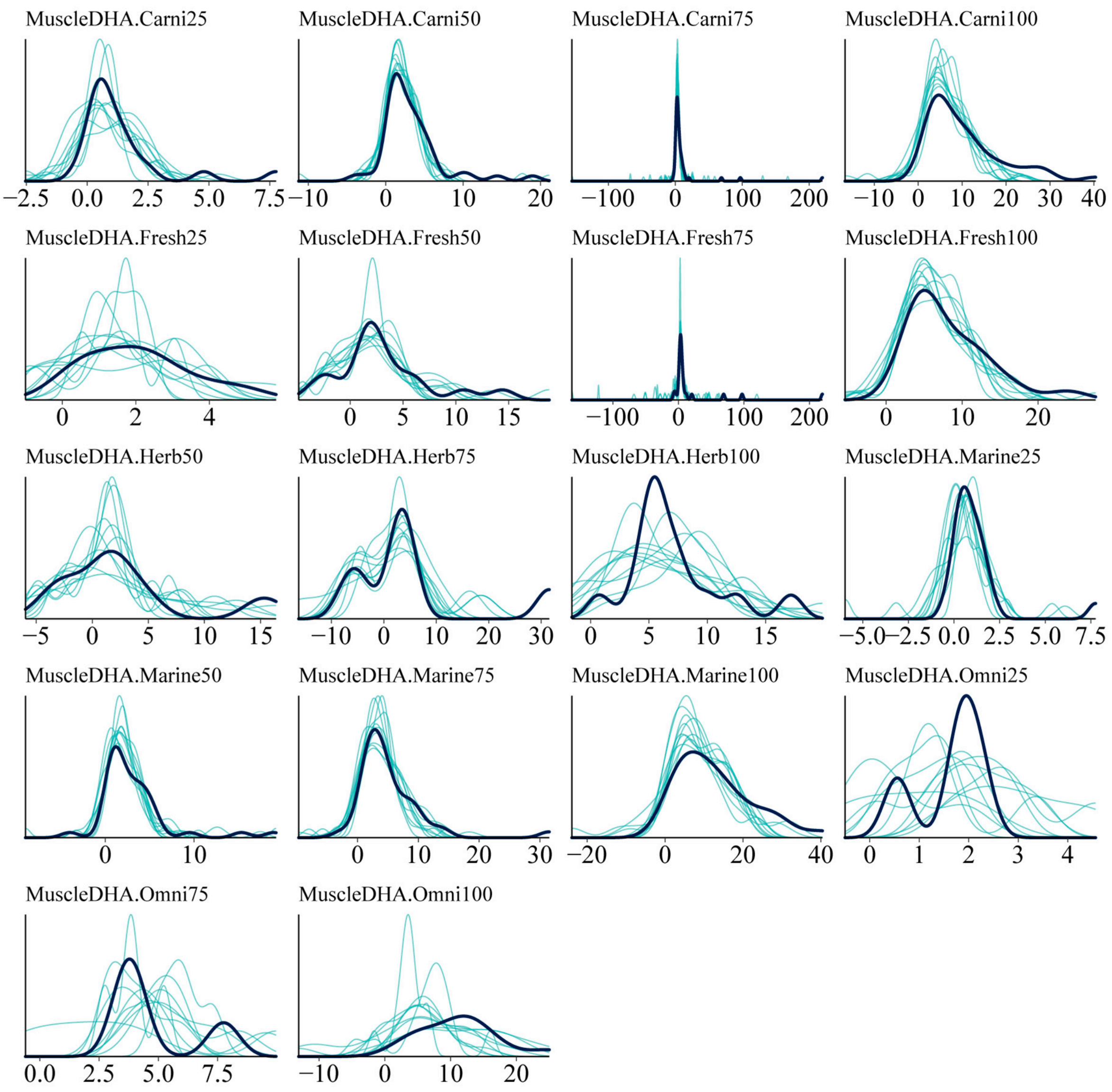

Figure S6. Posterior predictive check of simulated muscle DHA from the posterior predictive distribution. The 10 light blue lines represent the density distribution data of 10 simulated muscle DHA effect sizes randomly drawn from the posterior predictive distribution. The dark blue line represents the actual density distribution of muscle DHA effect sizes encompassed in this study. If the assumed model is convergent and valid, the simulated density distribution should roughly resemble the actual distribution. Herb, herbivorous fish; Omni, omnivorous fish; Carn, carnivorous fish; Fresh, freshwater fish; Marine, marine fish; DHA, docosahexaenoic acid; 25, 0 < RL ≤ 25; 50, 25 < RL ≤ 50; 75, 50 < RL ≤ 75; 100, 75 < RL ≤ 100.

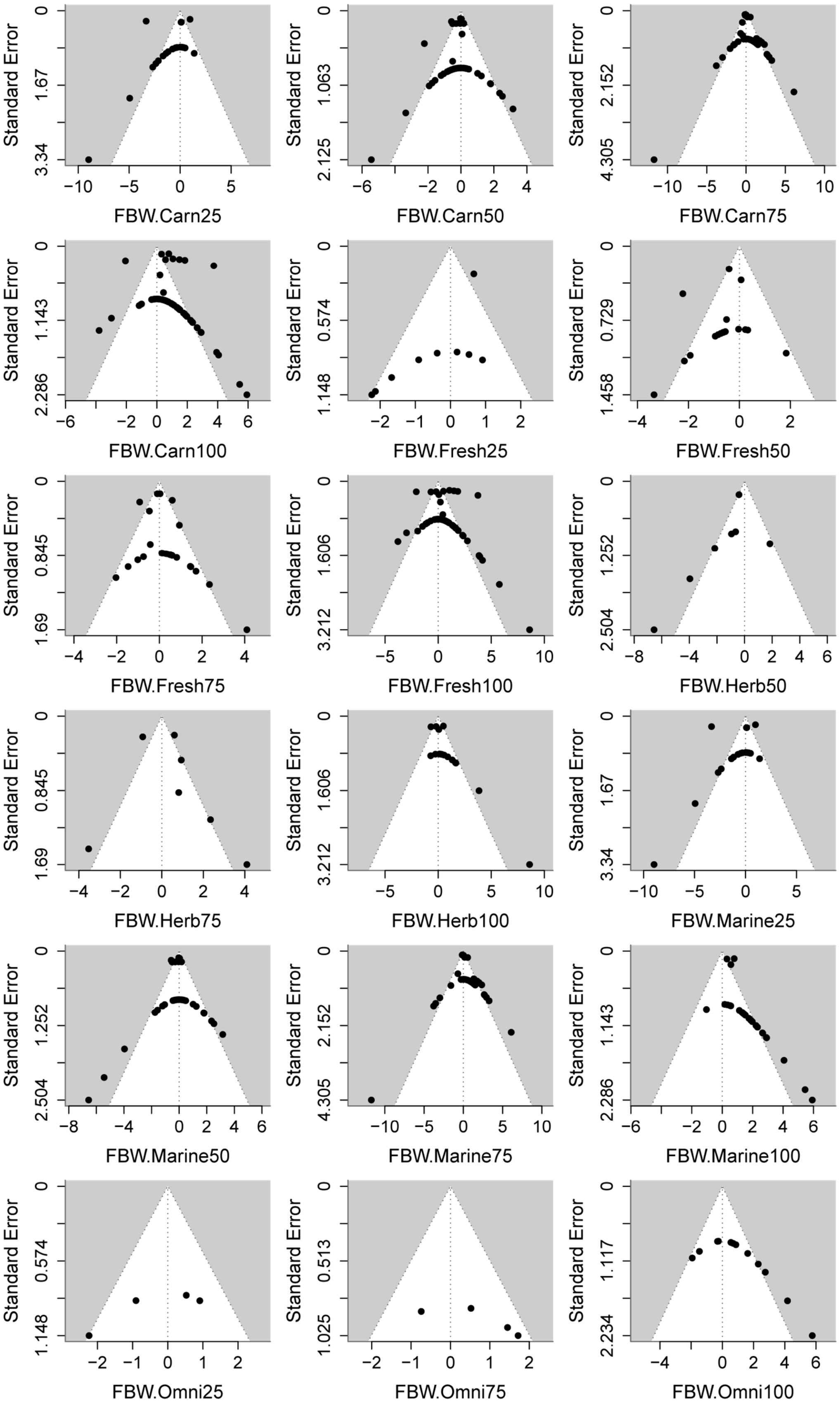

Figure S7. Funnel plot of FBW. The horizontal and vertical axes represent effect sizes and standard errors, respectively. FBW, final body weight; Herb, herbivorous fish; Omni, omnivorous fish; Carn, carnivorous fish; Fresh, freshwater fish; Marine, marine fish; 25, 0 < RL ≤ 25; 50, 25 < RL ≤ 50; 75, 50 < RL ≤ 75; 100, 75 < RL ≤ 100.

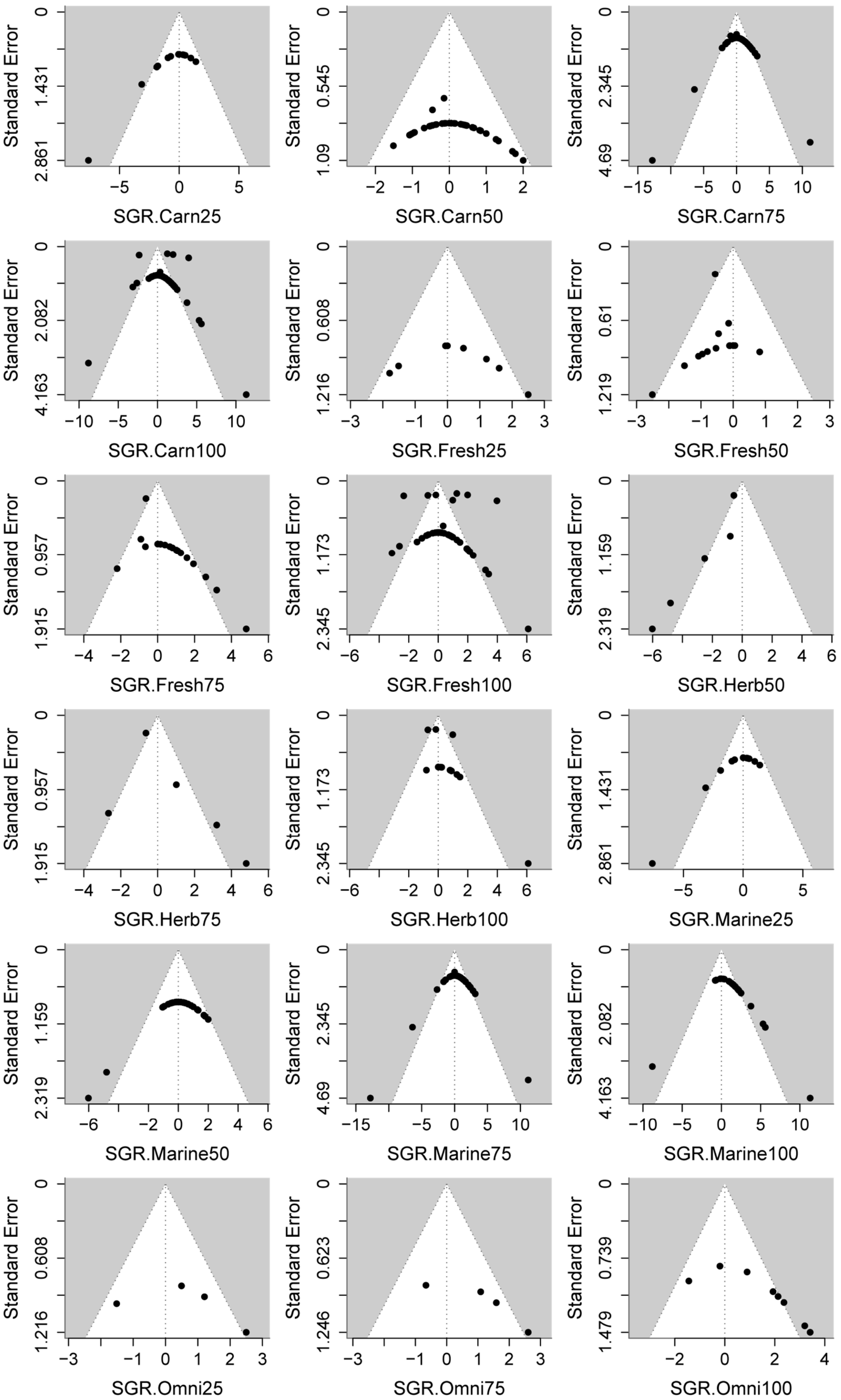

Figure S8. Funnel plot of SGR. The horizontal and vertical axes represent effect sizes and standard errors, respectively. SGR, specific growth rate; Herb, herbivorous fish; Omni, omnivorous fish; Carn, carnivorous fish; Fresh, freshwater fish; Marine, marine fish; 25, 0 < RL ≤ 25; 50, 25 < RL ≤ 50; 75, 50 < RL ≤ 75; 100, 75 < RL ≤ 100.

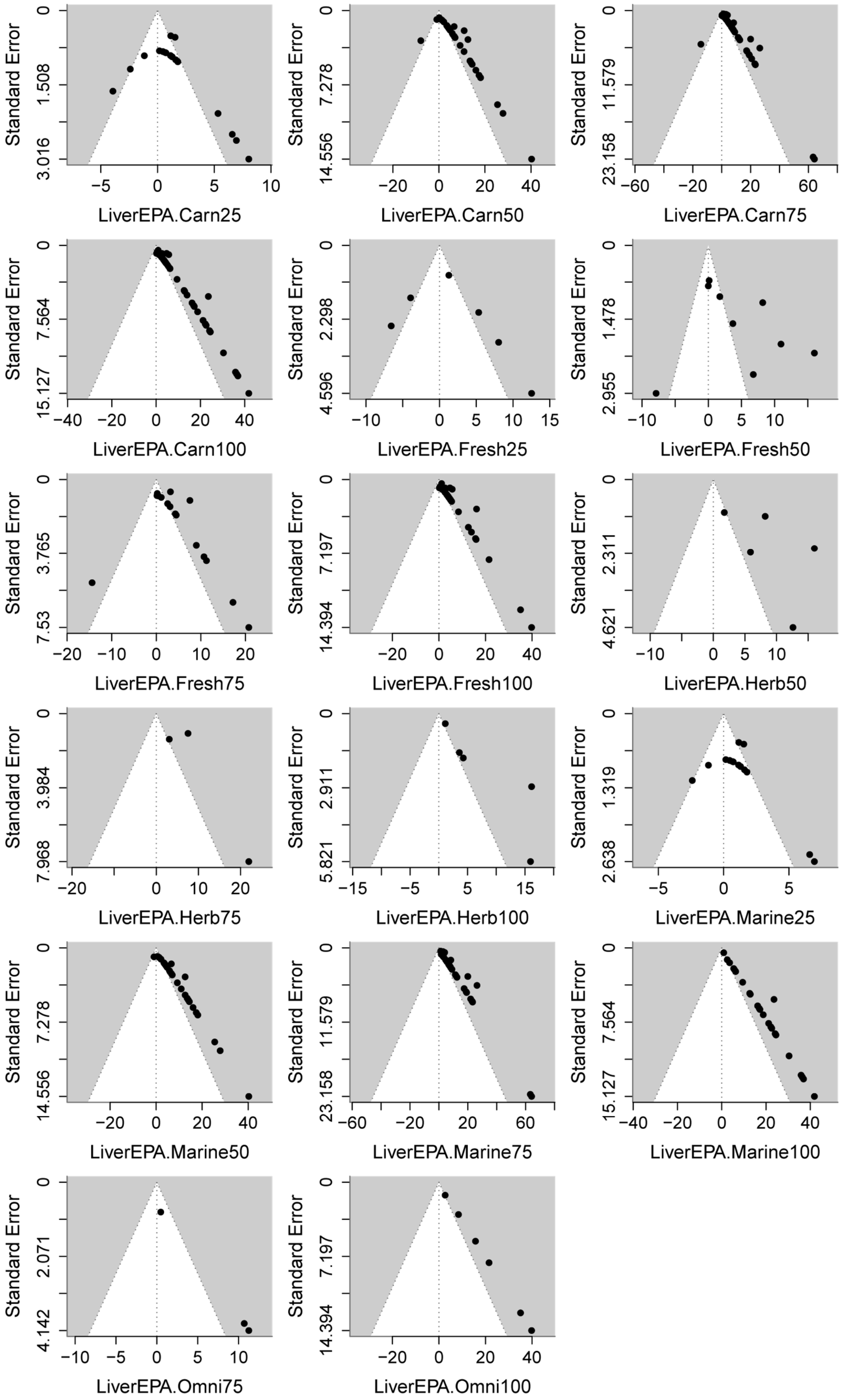

Figure S9. Funnel plot of liver EPA. The horizontal and vertical axes represent effect sizes and standard errors, respectively. Herb, herbivorous fish; Omni, omnivorous fish; Carn, carnivorous fish; Fresh, freshwater fish; Marine, marine fish; EPA, eicosapentaenoic acid; 25, 0 < RL ≤ 25; 50, 25 < RL ≤ 50; 75, 50 < RL ≤ 75; 100, 75 < RL ≤ 100.

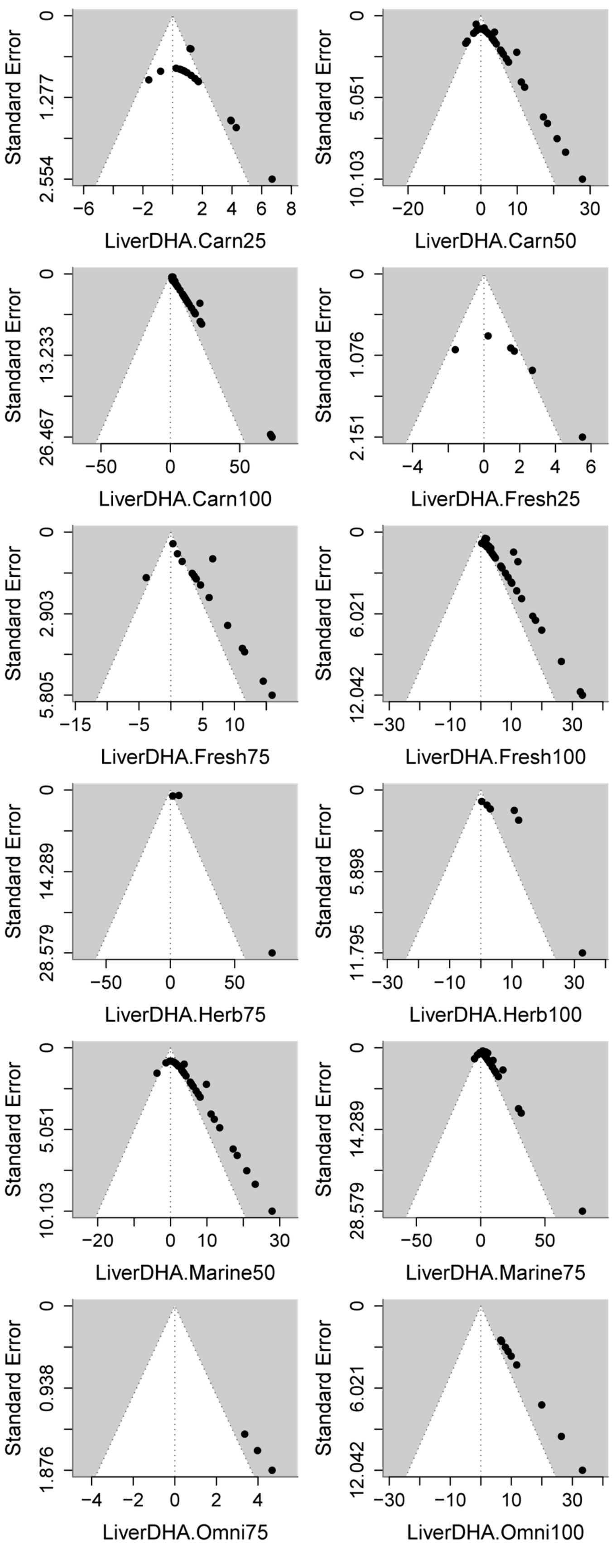

Figure S10. Funnel plot of liver DHA. The horizontal and vertical axes represent effect sizes and standard errors, respectively. Herb, herbivorous fish; Omni, omnivorous fish; Carn, carnivorous fish; Fresh, freshwater fish; Marine, marine fish; DHA, docosahexaenoic acid; 25, 0 < RL ≤ 25; 50, 25 < RL ≤ 50; 75, 50 < RL ≤ 75; 100, 75 < RL ≤ 100.

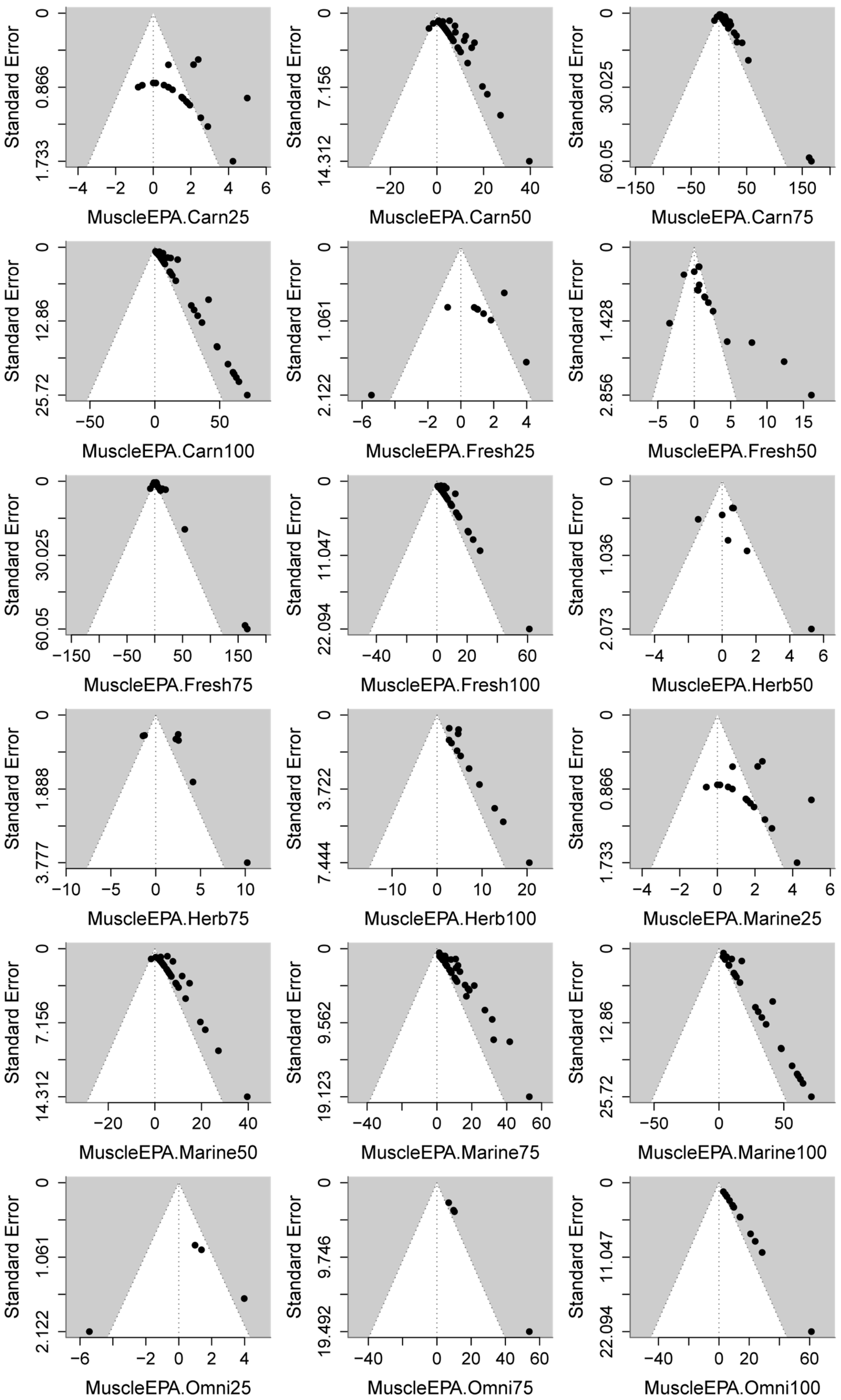

Figure S11. Funnel plot of muscle EPA. The horizontal and vertical axes represent effect sizes and standard errors, respectively. Herb, herbivorous fish; Omni, omnivorous fish; Carn, carnivorous fish; Fresh, freshwater fish; Marine, marine fish; EPA, eicosapentaenoic acid; 25, 0 < RL ≤ 25; 50, 25 < RL ≤ 50; 75, 50 < RL ≤ 75; 100, 75 < RL ≤ 100.

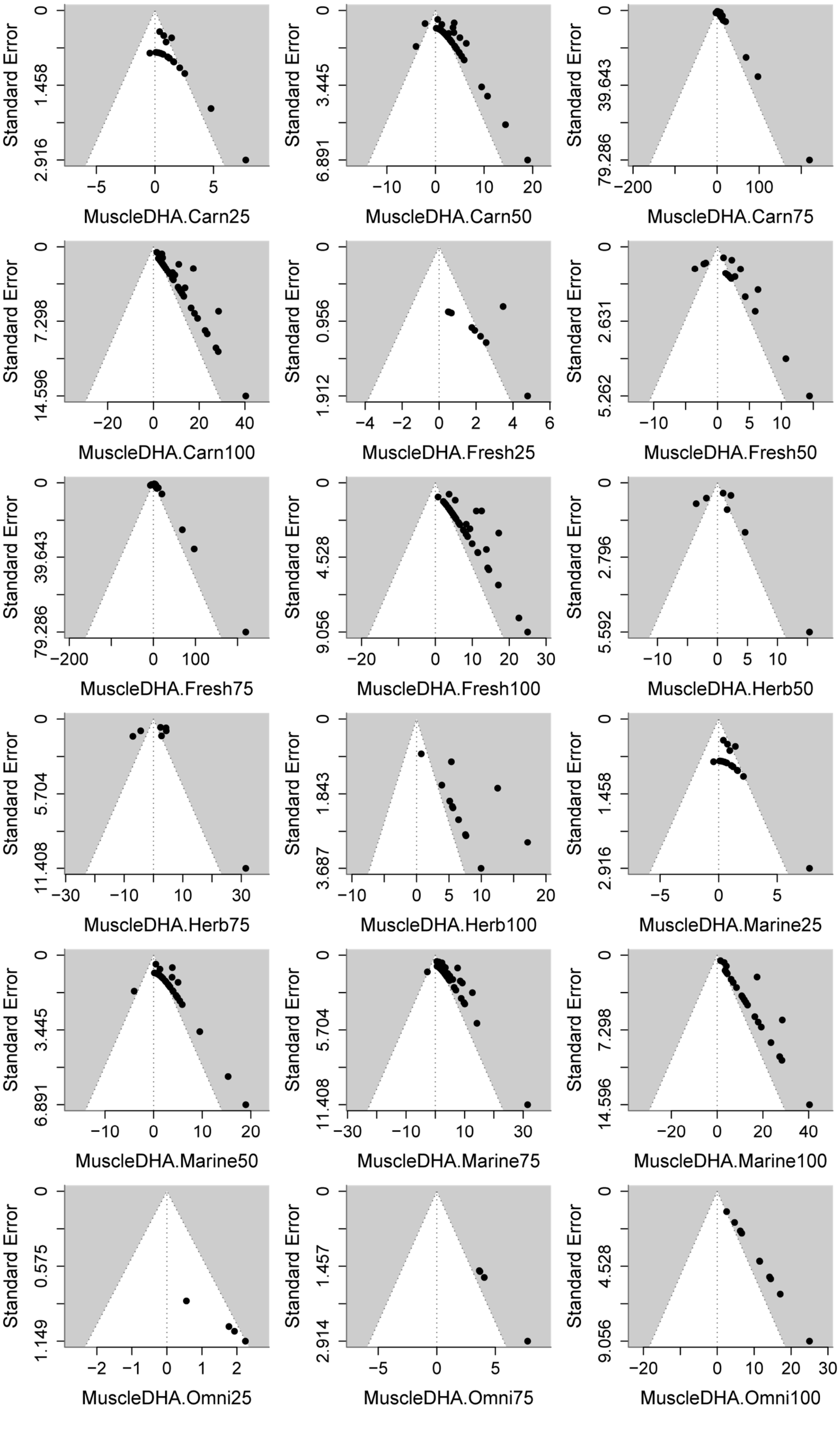

Figure S12. Funnel plot of muscle DHA. The horizontal and vertical axes represent effect sizes and standard errors, respectively. Herb, herbivorous fish; Omni, omnivorous fish; Carn, carnivorous fish; Fresh, freshwater fish; Marine, marine fish; DHA, docosahexaenoic acid; 25, 0 < RL ≤ 25; 50, 25 < RL ≤ 50; 75, 50 < RL ≤ 75; 100, 75 < RL ≤ 100.
